# Clusterin deficiency is associated with a lack of response to teriflunomide in multiple sclerosis

**DOI:** 10.1002/ctm2.1654

**Published:** 2024-04-09

**Authors:** Sunny Malhotra, Nicolas Fissolo, Carmen Rodríguez‐Rivera, Enric Monreal, Marta Montpeyo, Elena Urcelay, Juan Carlos Triviño, María José Pérez‐García, Miguel F. Segura, Agustín Pappolla, Jordi Río, Andreu Vilaseca, José Ignacio Fernández Velasco, Andrés Miguez, Carlos Goicoechea, Marta Martinez‐Vicente, Luisa M Villar, Xavier Montalban, Manuel Comabella

**Affiliations:** ^1^ Servei de Neurologia. Centre d'Esclerosi Múltiple de Catalunya (Cemcat). Institut de Recerca Vall d'Hebron (VHIR). Hospital Universitari Vall d'Hebron. Universitat Autònoma de Barcelona Barcelona Spain; ^2^ Area of Pharmacology and Nutrition & Bromatology Department of Basic Health Sciences Universidad Rey Juan Carlos, Alcorcón Madrid Spain; ^3^ Department of Neurology Hospital Universitario Ramón y Cajal REEM IRYCIS Universidad de Alcalá Madrid Spain; ^4^ Neurodegenerative Diseases Research Group, Institut de Recerca Vall d'Hebron (VHIR) ‐ Center for Networked Biomedical Research on Neurodegenerative Diseases (CIBERNED) Barcelona Spain; ^5^ Laboratorio de Investigación en Genética de Enfermedades Complejas Instituto de Investigación Sanitaria San Carlos (IdISSC) Madrid Spain; ^6^ Genomic Systems Valencia Spain; ^7^ Group of Childhood Cancer and Blood Disorders Institut de Recerca Vall d'He bron (VHIR) Universitat Autònoma de Barcelona Barcelona Spain

Dear Editor,

We identified clusterin (CLU) as a treatment response biomarker to teriflunomide, a finding that will contribute to personalized treatment in multiple sclerosis (MS) patients.

Teriflunomide, an orally administered therapy for relapsing‐remitting MS (RRMS) patients that inhibits the mitochondrial enzyme dihydroorotate dehydrogenase, does not produce clinical benefits in some patients. To identify teriflunomide treatment response biomarkers, we performed RNA sequencing (RNA‐seq) in peripheral blood mononuclear cells (PBMCs) from a first cohort of RRMS patients classified into responders and non‐responders after 12 months of treatment^1^ (Table S1). Genes showing differential expression (adjusted *p*‐values < .001) were selected for polymerase chain reaction (PCR) validation (Figure 1A,B). The remaining genes are shown in Table S2 and the baseline differences of selected genes in Figure S1. Considering the antiviral effects of teriflunomide^2^ and the functional enrichment observed for type I interferon (IFN) categories (Figure 1C), additional type I IFN‐related genes were included for PCR validation (Figure 1D). Validated genes (Figure 1E and Figures S2 and S3) were measured in a second and independent cohort of RRMS patients (Table S3). Only CLU was validated and expression levels were downregulated in non‐responders after 12 months of treatment (Figure 1F and Figures S4 and S5). Interestingly, differential downregulation of CLU in non‐responders after treatment was specific to teriflunomide, since CLU expression levels in non‐responders to other oral therapies such as dimethyl fumarate or fingolimod were not reduced after treatment (Table S4 and Figure 1G). Based on our RNA‐seq findings pointing to CLU as a teriflunomide treatment response biomarker, we next performed PBMC immunophenotyping (Table S5) to identify the cell population responsible for the decreased CLU expression in non‐responders. Clusterin expression was primarily reduced in CD4^+^ and CD8^+^ T cells from non‐responders after treatment (Figure 2A–C).

**FIGURE 1:**
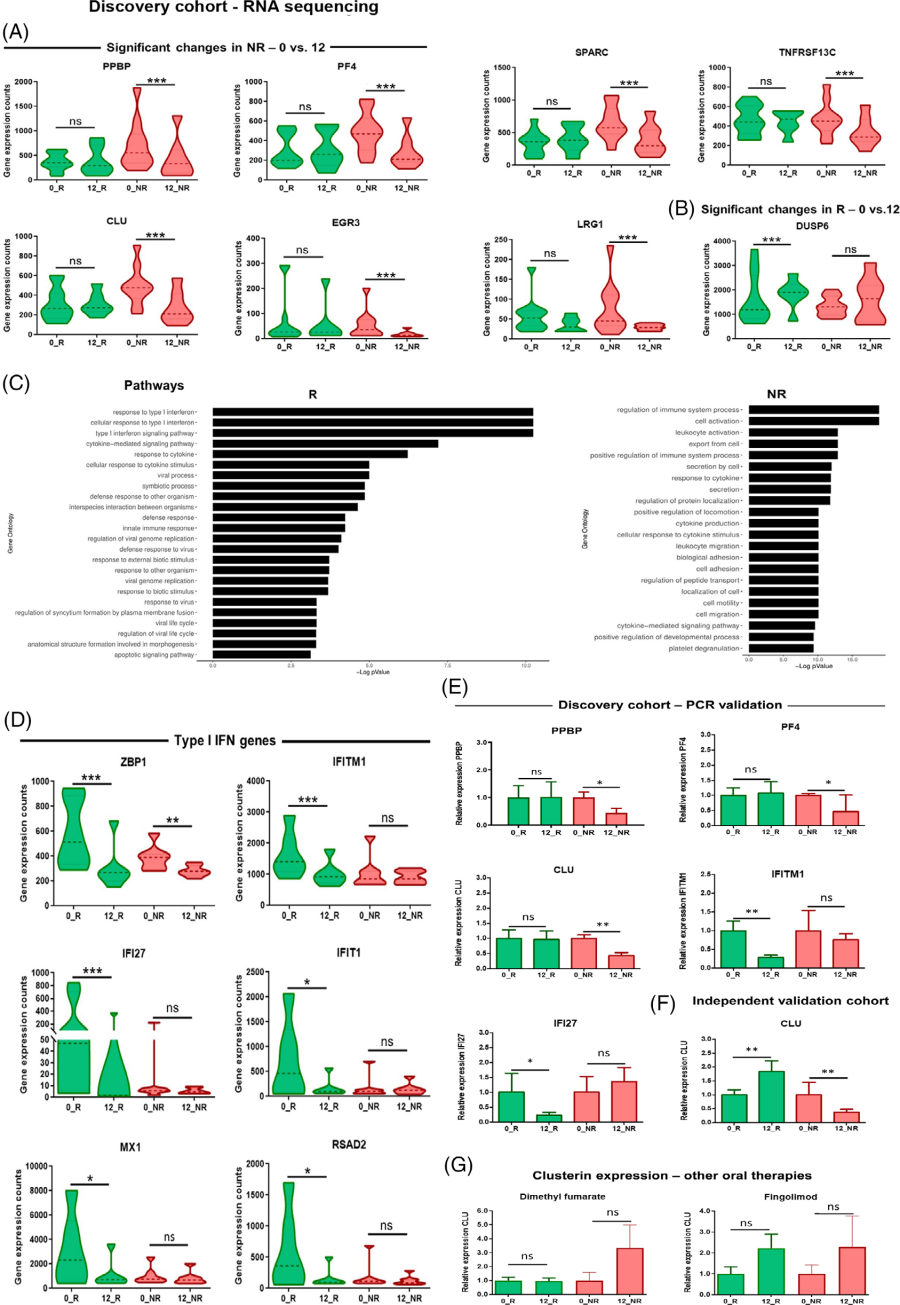
Violin plots representing expression levels of selected genes from the RNA sequencing in peripheral blood mononuclear cells (PBMCs) from responders and non‐responders to teriflunomide based on the differential expression between the baseline and the 1‐year treated conditions. Classification of patients into responders and non‐responders was performed according to the Rio score^1^ after 12 months of treatment with teriflunomide. Non‐responders were patients fulfilling at least one of the following criteria: ≥1 relapse, ≥3 new or active lesions in the magnetic resonance imaging (MRI), and progression of ≥1 point in the Expanded Disability Status Scale (EDSS) in comparison to baseline clinical and MRI data. Patients with no relapses, no EDSS progression, and < 3 new or active lesions in the 1‐year MRI were classified as responders. (A) Differentially expressed genes in non‐responders but not in responders with adjusted *p*‐values < .001. (B) Differentially expressed genes in responders but not in non‐responders with adjusted *p*‐values < .001. (C) Relevant pathways in teriflunomide responders and non‐responders with adjusted *p*‐values < .05. (D) Type I interferon genes selected for real‐time polymerase chain reaction (PCR) validation. (E) Expression levels of genes validated by real‐time PCR in the discovery cohort. (F) Clusterin expression levels in an independent validation cohort of relapsing‐remitting multiple sclerosis (RRMS) patients treated with teriflunomide. (G) Clusterin expression levels in RRMS patients treated with dimethyl fumarate and fingolimod. Bars and whiskers in (E–G) represent the mean and standard error of the mean. 0_R/0_NR: baseline expression levels in responders and non‐responders, respectively. 12_R/12_NR: expression levels after 12 months of teriflunomide treatment in responders and non‐responders, respectively. DUSP6: dual specificity phosphatase 6. CLU: clusterin. EGR3: early growth response 3. IFI27: interferon alpha inducible protein 27. IFITM1: interferon‐induced transmembrane protein 1. IFIT1: interferon‐induced protein with tetratricopeptide repeats 1. LRG1: leucine rich alpha‐2‐glycoprotein 1. MX1: MX dynamin‐like GTPase 1. PF4: platelet factor 4. PPBP: pro‐platelet basic protein. SPARC: secreted protein acidic and cysteine‐rich. TNFRSF13C: TNF receptor superfamily member 13C. RSAD2: radical S‐adenosyl methionine domain containing 2. ZBP1: Z‐DNA binding protein 1. *p < .05, **p < .01, ***p < .001. ns: *p* > .05.

**FIGURE 2:**
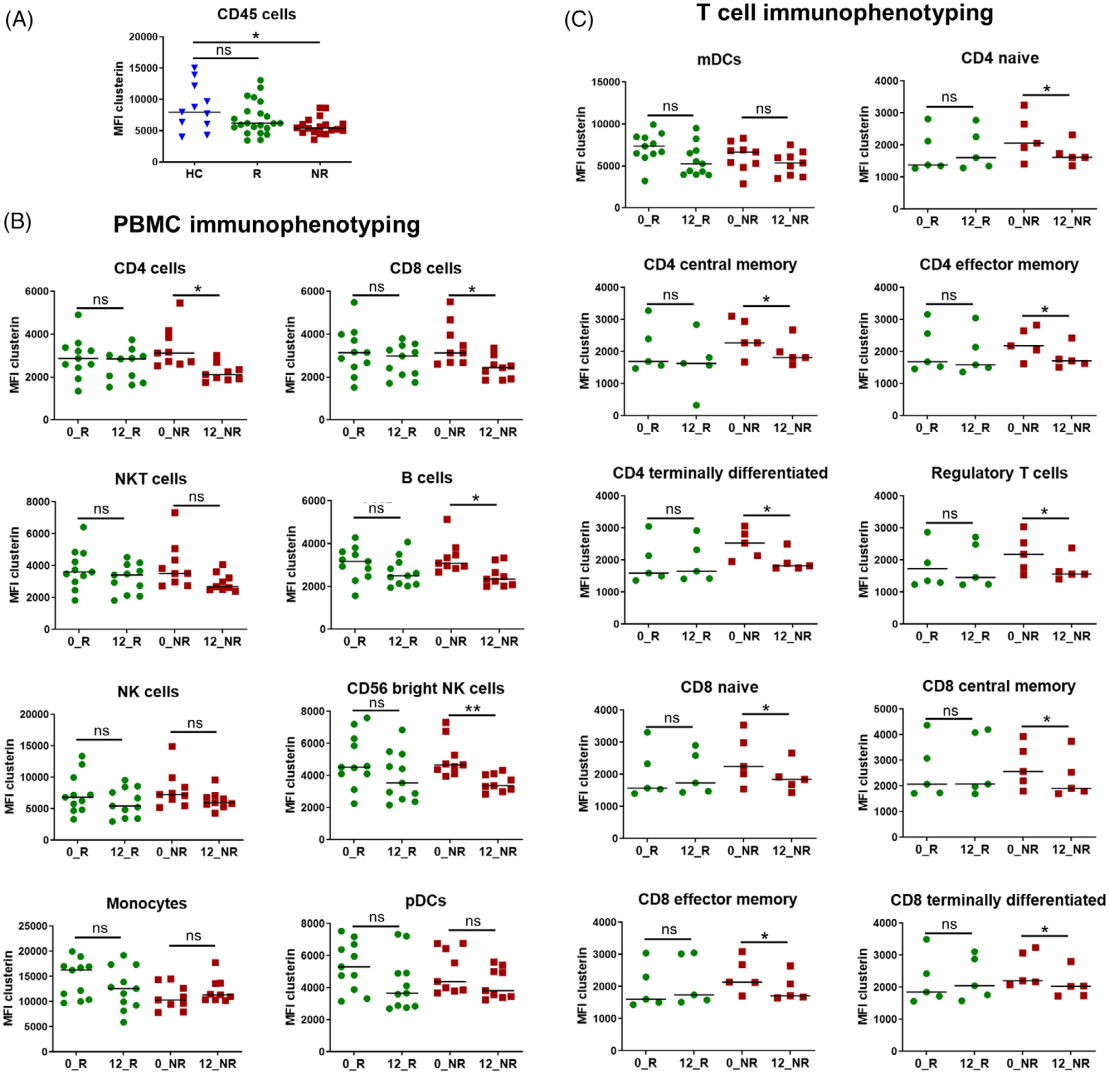
Clusterin expression determined by flow cytometry in different peripheral blood cell populations. (A) Clusterin expression in peripheral blood mononuclear cells (PBMCs) (CD45+) of healthy controls (HC; *N* = 11; seven females/four males; mean age (standard deviation): 41.3 (5.5) years) and the whole group of responders (R; *N* = 11) and non‐responders (NR; *N* = 9). Both R and NR include the baseline and teriflunomide‐treated conditions. Clusterin expression is decreased in CD45+ cells of relapsing‐remitting multiple sclerosis (RRMS) patients compared to HC, and differences reached statistical significance when compared to non‐responders. (B) Clusterin expression in different PBMC populations. Boxplots reveal preferential reduction in clusterin expression in CD3 expressing T cells (CD4^+^ and CD8^+^) from non‐responders after 12 months of treatment. A trend was also observed for NKT cells (*p* = .05). Although clusterin expression was decreased in B cells and CD56^bright^ NK cells from non‐responders after treatment, a trend towards a significant decrease in clusterin expression was also observed in responders (*p* = .06 for both blood cell populations). (C) Clusterin expression in different T cell populations. Boxplots reveal a similar pattern of significant reduction in clusterin expression in all T cell populations from non‐responders by the effect of treatment. Each symbol represents an individual and horizontal bars indicate median values. NK: natural killer. mDCs: myeloid dendritic cells. pDCs: plasmacytoid dendritic cells. PBMC: peripheral blood mononuclear cells. 0_R/0_NR: clusterin expression levels at baseline in responders and non‐responders, respectively. 12_R/12_NR: clusterin expression levels after 12 months of teriflunomide treatment in responders and non‐responders, respectively. MFI: mean fluorescence intensity. *p*‐Values were obtained after comparisons between groups by means of a Mann‐Whitney U test. *p < .05, **p < .01. ns: *p* > .05.

Clusterin is a stress‐induced multifunctional molecular chaperone expressed as a partially glycosylated presecreted isoform and also as a fully glycosylated mature isoform ready to be secreted outside the cell.^3^ Based on this, we next investigated intracellular CLU expression in the cytosolic and mitochondrial fractions of PBMC from patients (Figure S6). Teriflunomide treatment was associated with decreased expression of the cytosolic mature secreted CLU isoform and increased expression of the mitochondrial presecreted CLU isoform in non‐responders (Figure 3A). Investigation of other CLU‐interacting proteins revealed increased cytosolic bax expression in non‐responders after treatment, which was paired with reduced mitochondrial bax expression and increased mitochondrial cytochrome C expression both in non‐responders after treatment (Figure 3B,C). Cleaved‐caspase 3 expression in resting cells was decreased in non‐responders following treatment, though differences were not statistically significant (Figure 3D). Noteworthy, the expression pattern of CLU isoforms detected in non‐responders after treatment was more prominent at 12 months since it was not observed at earlier time points (Figure 3E). Since CLU has been reported to mediate processes such as endoplasmic reticulum (ER) stress and autophagy,^4^ we also explored whether teriflunomide had the potential to induce ER stress and autophagy. In‐vitro exposure of PBMC from controls to teriflunomide was associated with increased expression of BiP and LC3‐II (Figure 3F). However, expression levels for BiP and LC3‐II were comparable at baseline between responders and non‐responders (Figure 3G), indicating that our CLU‐related findings are triggered by teriflunomide treatment.

**FIGURE 3:**
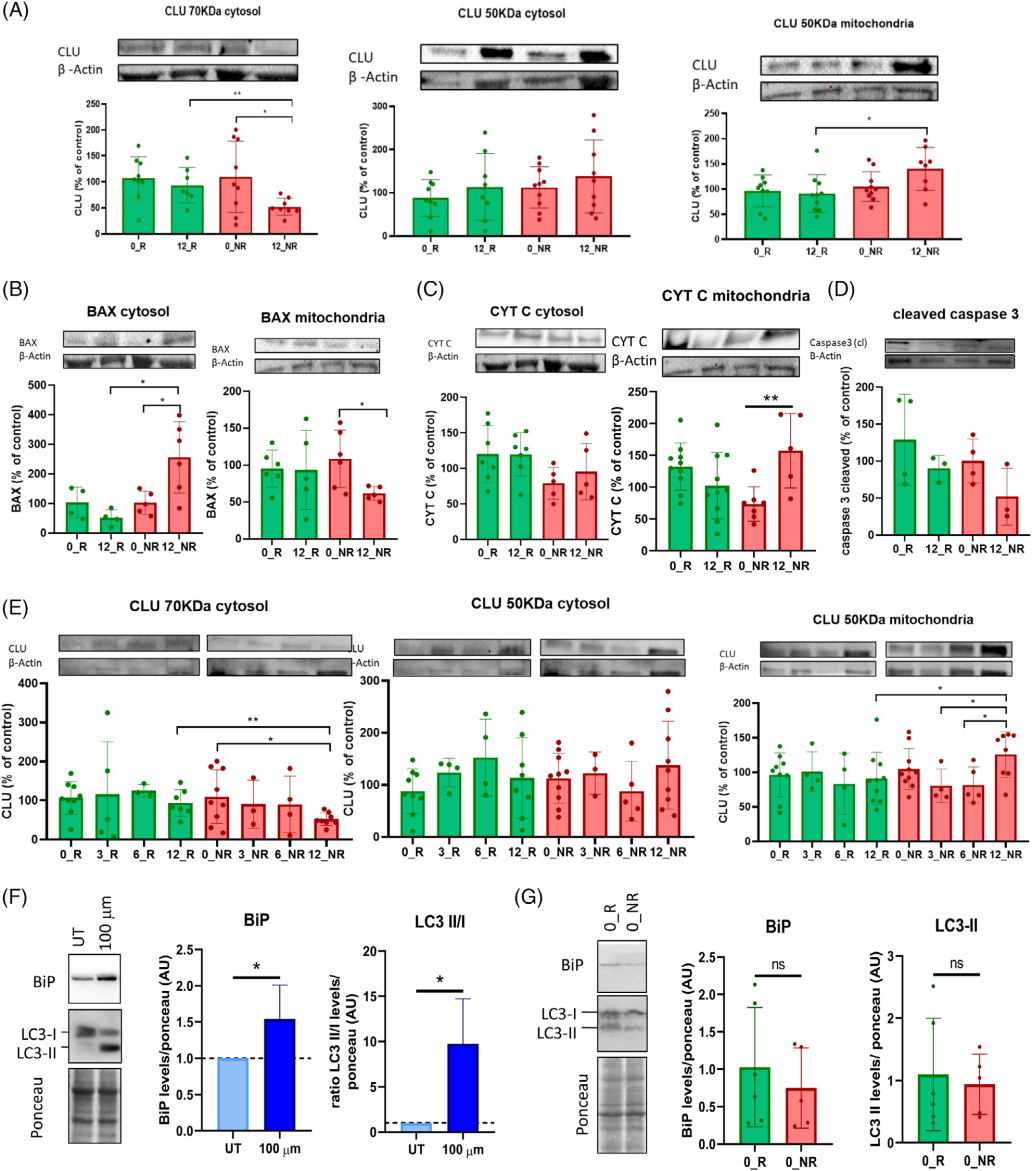
Expression of the different clusterin isoforms determined by western blot in the cytosolic and mitochondrial fractions of peripheral blood mononuclear cells (PBMCs) from responders (*N* = 10) and non‐responders (*N* = 10) to teriflunomide. (A) Expression of the clusterin isoforms in the cytosolic and mitochondrial fractions. In the cytosol, the mature secreted clusterin isoform was significantly decreased after treatment in teriflunomide non‐responders compared to responders and also compared to the baseline untreated condition, whereas expression of the 50 KDa presecreted clusterin isoform was similar across the different groups. In contrast, expression of the presecreted clusterin isoform in the mitochondria was significantly increased in non‐responders versus responders after treatment, and a trend was also observed when compared to the baseline in non‐responders (*p* = .05). (B, C) Expression of BAX and CYT C in the cytosolic and mitochondrial fractions, respectively. Cytosolic bax expression was significantly increased in non‐responders after 12 months of treatment compared to responders and also compared to the baseline, and mitochondrial bax expression was significantly reduced also in non‐responders after treatment compared to the baseline. Cytochrome C expression in the mitochondrial fraction was significantly increased in non‐responders after treatment compared to the untreated condition, whereas expression in the cytosolic fraction was comparable across the different groups. (D) Cleaved‐caspase 3 expression in resting (unstimulated) PBMC. Expression was reduced in non‐responders by the effect of treatment but differences were not statistically significant. (E) Expression of clusterin isoforms at different time points during the first year of teriflunomide treatment (baseline, 3, 6, and 12 months). Changes in the expression of clusterin isoforms are more prominent after 12 months. (F) Expression of BiP (as a marker of endoplasmic reticulum stress) and LC3‐II (as a marker of autophagy) after in vitro exposure to teriflunomide (100 µM for 24 h) in whole PBMC from healthy controls (*N* = 6; three females/three males; mean age [standard deviation]: 24.3 [3.7] years). (G) Expression of BiP and LC3‐II in whole PBMC from responders and non‐responders at baseline. No significant differences are observed between groups. For all graphs, bars and whiskers represent the mean and standard deviation. 0_R/0_NR: baseline expression in responders and non‐responders, respectively. 3_R/3_NR: expression after 3 months of teriflunomide treatment in responders and non‐responders, respectively. 6_R/6_NR: expression after 6 months of teriflunomide treatment in responders and non‐responders, respectively. 12_R/12_NR: expression after 12 months of teriflunomide treatment in responders and non‐responders, respectively. UT: untreated cells. BAX: BCL2 associated X, apoptosis regulator. CLU: clusterin. CYT C: cytochrome C. *p*‐Values were obtained after comparisons between groups by means of a Mann‐Whitney U test. *p < .05, **p < .01.

Teriflunomide treatment was reported to reduce the T‐cell respiratory and glycolytic capacities in RRMS patients^5^; however, we did not observe significant differences in the T‐cell metabolic profile between teriflunomide responders and non‐responders (Figure 4A,B and Figure S7).

**FIGURE 4:**
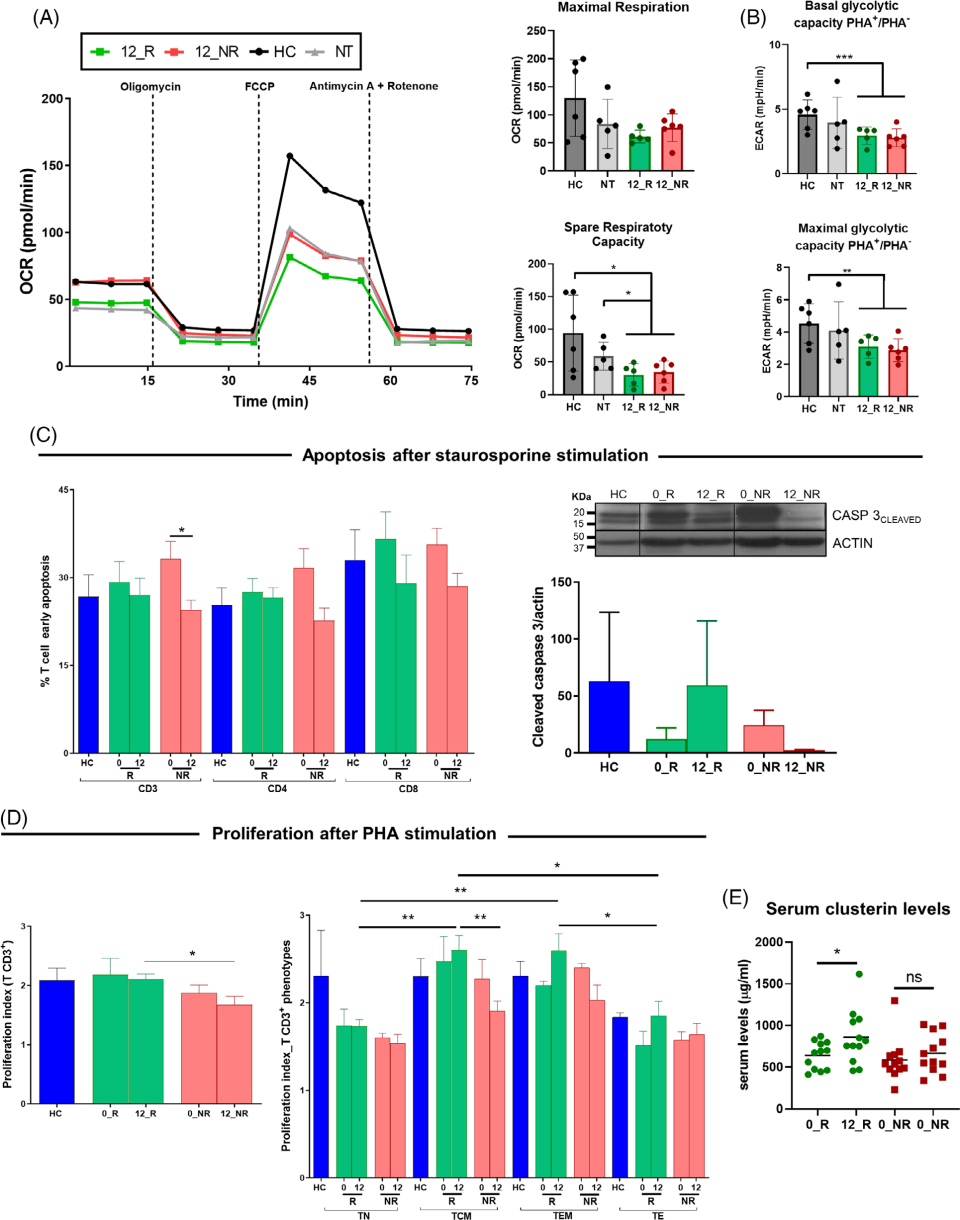
Metabolic profile, apoptosis and proliferation of peripheral blood mononuclear cells (PBMCs) from teriflunomide responders and non‐responders. (A) Oxygen consumption rate kinetics, maximal respiration and spare respiratory capacity, and (B) basal and maximal glycolytic capacity (ratio in the presence/absence of PHA) of PBMC from healthy controls (*N* = 6), untreated MS patients (*N* = 5), and teriflunomide responders (*N* = 5) and non‐responders (*N* = 6). T cell metabolism was analyzed in a Seahorse XFp extracellular flux analyser. Spare respiratory capacity was reduced in teriflunomide responders and non‐responders compared to either untreated MS patients or controls, and basal and maximal glycolytic capacities were also decreased in teriflunomide‐treated patients regardless of their response status when compared to healthy controls. Bars and whiskers represent the mean and standard deviation. (C) To study apoptosis, PBMC from responders (*N* = 6) and non‐responders (*N* = 5) after 12 months of teriflunomide treatment were stimulated with 2 µM of staurosporine, or left untreated for 16 h in a complete medium. PBMC from responders (N = 3) and non‐responders (*N* = 3) at baseline, and healthy controls (*N* = 4) stimulated in vitro with teriflunomide (100 µM) for 24 h were also included for comparison purposes. Left: bars showing early apoptosis of T cells. Apoptosis was determined by means of Annexin V/7AAD staining using the FlowJo software in CD3^+^, CD3^+^CD4^+^, and CD3^+^CD8^+^ T cells. Percentages of gated CD3^+^, CD3^+^CD4^+^ and CD3^+^CD8^+^ that show positivity for Annexin V and negativity for 7AAD (early apoptotic) are represented. Early apoptosis in CD3^+^ T cells (Annexin V^+^/7AAD^−^) was significantly reduced in non‐responders after teriflunomide treatment compared to the baseline, and trends towards decreased early apoptosis were also observed for CD4^+^ T cells (*p* = .05) and CD8^+^ T cells (*p* = .09) compared to baseline. Right: western blot images of cleaved‐caspase 3 in whole PBMC after staurosporine stimulation. Representative image of cleaved‐caspase 3 and quantitative analysis using Image J. A trend towards decreased expression of cleaved‐caspase 3 was observed in non‐responders after 12 months of teriflunomide treatment compared to the untreated condition (*p* = .05). (D) Proliferation was assessed by measuring carboxyfluorescein succinimidyl ester (CFSE) dilution using flow cytometry. Bars showing the proliferation index in CFSE‐stained T cells from PBMC from responders (*N* = 6) and non‐responders (*N* = 5) after 12 months of teriflunomide treatment, responders (*N* = 3) and non‐responders (*N* = 3) at baseline, and healthy controls (*N* = 4). Cells were stimulated with PHA at 0.5 µg/ml and labelled with CFSE. After 7 days, the proliferation index was determined using the FlowJo software in CD3^+^ T cells, and in different T cell phenotypes following a combination of CCR7/CD45RA staining. The proliferative response of memory T cell populations was reduced in non‐responders by the effect of teriflunomide, and differences reached statistical significance for central memory T cells and remained as a trend for effector memory T cells (*p* = 0.06) compared to responders. Results in (C) and (D) are given as mean and standard error of the mean. (E) Serum clusterin levels were determined by enzyme‐linked immunosorbent assay (ELISA) before and after teriflunomide treatment in responders (*N* = 12) and non‐responders (*N* = 12). Clusterin levels were significantly elevated in responders after 12 months of treatment compared to the baseline, whereas clusterin levels in non‐responders were not modified by the effect of treatment. Each symbol represents an individual and horizontal bars indicate mean values. OCR: Oxygen Consumption Rate. ECAR: Extracellular Acidification Rate. HC: healthy controls. NT: untreated MS patients. TN: naive T cells. TCM: central memory T cells. TEM: effector memory T cells. TE: terminally differentiated T cells. 0_R/0_NR: baseline levels of parameters in responders and non‐responders, respectively. 12_R/12_NR: baseline parameters after 12 months of teriflunomide treatment in responders and non‐responders, respectively. *p < .05, **p < .01, ***p < .001. ns: *p* > .05.

Under conditions like ER stress, the presecreted isoform of CLU can bind to BiP, which stabilizes CLU and facilitates its retrotranslocation to the cytosol and redistribution to mitochondria,^6^ where CLU inhibits apoptosis.^7^ Furthermore, the mature secreted CLU isoform can modulate cell proliferation,^3^ a process inhibited by teriflunomide, particularly in T and B cells with high proliferative rates.^8^, ^9^ Considering these two CLU‐related properties, we evaluated whether the differential expression pattern of CLU isoforms observed between responders and non‐responders could translate into differences in the apoptotic and proliferation capacities of T cells from both groups of patients (Table S6 and Figure S8). Compared to baseline, early apoptosis in CD3^+^ T cells was reduced in non‐responders after treatment, and trends were found for CD4^+^ and CD8^+^ T cells (Figure 4C), whereas no differences were seen for T cells in late apoptosis/necrosis (Figure S9). A trend towards decreased expression of cleaved‐caspase 3 was observed in PBMC from non‐responders after treatment compared to the untreated condition (Figure 4C), altogether supporting a higher resistance of T cells from non‐responders to apoptosis after teriflunomide treatment. The proliferative response of CD3^+^ T cells was reduced after treatment in non‐responders compared to responders (Figure 4D). Proliferation indexes of memory T cell populations from responders after treatment were higher compared to the naive and terminally differentiated T cell populations (Figure 4D). Interestingly, these significant differences in the proliferation indexes were lost in non‐responders due to a selective reduction in the proliferative response of memory T cell populations by treatment, which reached statistical significance for central memory T cells and remained as a trend for effector memory T cells compared to responders (Figure 4D). Finally, since mature CLU isoform may egress cells and exert extracellular chaperone activities,^10^ we measured the serum levels of the mature secreted CLU isoform before and after treatment. Clusterin levels were elevated in responders after treatment (Figure 4E), reflecting the differences observed inside cells with lower levels in teriflunomide non‐responders.

In summary, teriflunomide treatment induces ER stress in blood cells, which in non‐responders is associated with redistribution of the presecreted CLU isoform to mitochondria, and decreased expression of the mature secreted isoform in the cytosol, particularly in CD3+ T cells. Increased mitochondrial presecreted CLU expression will result in higher survival of CD3+ T cells, including pathogenic T cell populations. Deficiency of mature secreted CLU isoform will result in reduced proliferation of memory CD3+ T cell populations, which will probably escape from the antiproliferative effects of teriflunomide, further contributing to their abnormal persistence in the peripheral blood of non‐responders. Although these findings need validation in future prospective cohorts of patients, they have implications in clinical practice to identify teriflunomide non‐responders early during treatment based on CLU expression changes in blood cells.

## CONFLICT OF INTEREST STATEMENT

The authors declare no conflict of interest.

## FUNDING INFORMATION

This research was conducted using Departmental funds for Medical Research.

## ETHICS STATEMENT

The study was approved by the local ethics committee and MS patients gave their informed consent to EPA(AG)57/2013(3834).

## Supporting information

Supporting Information

Supporting Information

Supporting Information

Supporting Information

Supporting Information

Supporting Information

Supporting Information

Supporting Information

Supporting Information

Supporting Information

Supporting Information

## Data Availability

The dataset used or analyzed for the current study is available from the corresponding author upon reasonable request.
