# Supplementary material for: Clusterin deficiency is associated with a lack of response to teriflunomide in multiple sclerosis
Source: Clin Transl Med. 2024 Apr 9;14(4):e1654. doi: 10.1002/ctm2.1654 (PMC11003271; doi:10.1002/ctm2.1654)

**Figure S1.** Violin plots showing baseline expression levels of genes selected from the RNA sequencing in responders (N=11) and non-responders (n=10) to teriflunomide. 0_R/0_NR: baseline expression levels in responders and non-responders, respectively. DUSP6: dual specificity phosphatase 6. CLU: clusterin. EGR3: early growth response 3. IFI27: interferon alpha inducible protein 27. IFITM1: interferon induced transmembrane protein 1. IFIT1: interferon induced protein with tetratricopeptide repeats 1. LRG1: leucine rich alpha-2-glycoprotein 1. MX1: MX dynamin like GTPase 1. PF4: platelet factor 4. PPBP: pro-platelet basic protein. SPARC: secreted protein acidic and cysteine rich. TNFRSF13C: TNF receptor superfamily member 13C. RSAD2: radical S-adenosyl methionine domain containing 2. ZBP1: Z-DNA binding protein 1. P-values were obtained after comparisons between groups by means of a Mann-Whitney U test. *P<0.05, **p<0.01, ns: p>0.05.

**
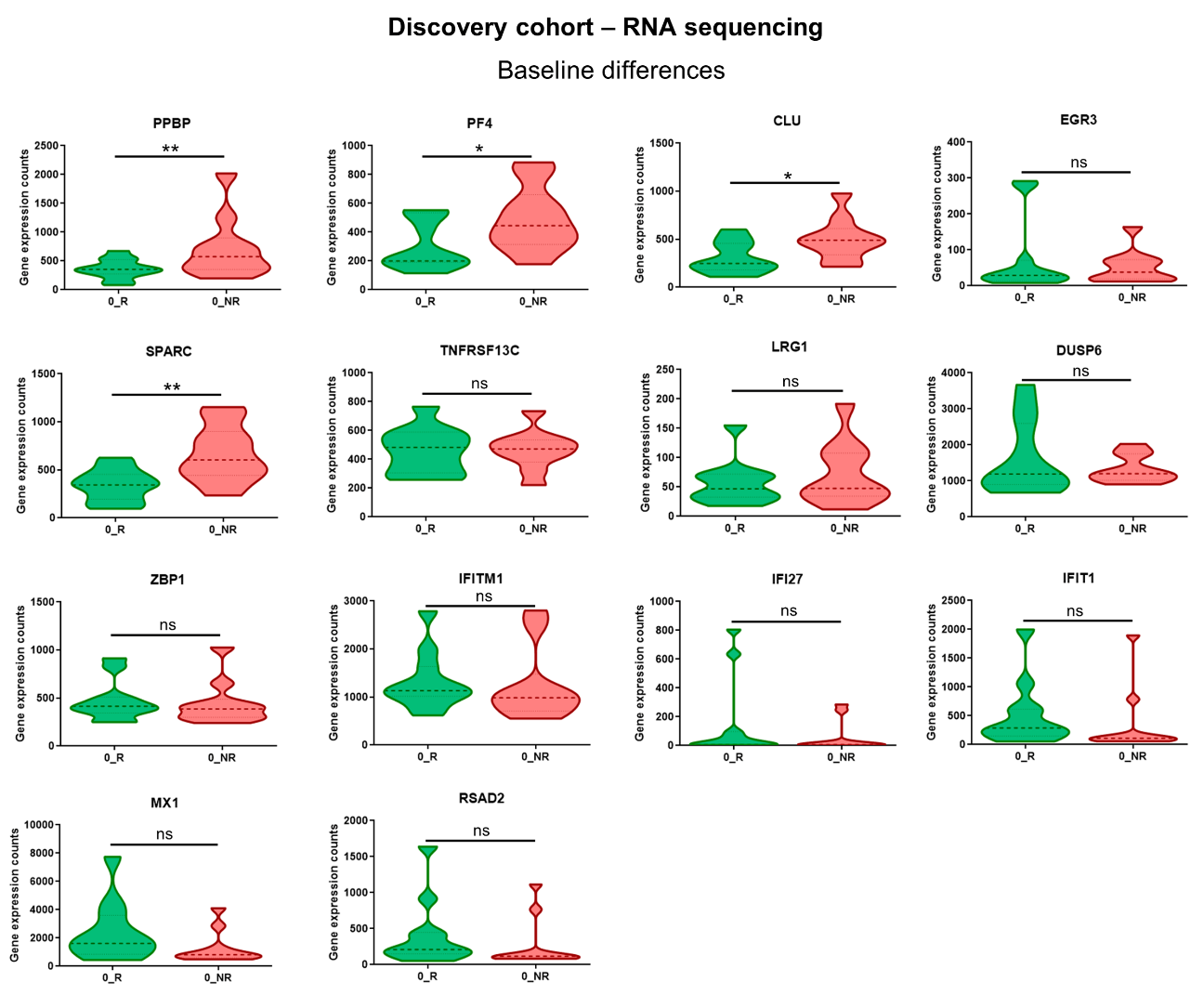
**

**Figure S2.** Expression levels of genes selected from the RNA sequencing in PBMC from responders and non-responders to teriflunomide that were not validated by real-time PCR in the discovery cohort. Bars and whiskers represent mean and standard error of the mean. 0_R/0_NR: baseline expression levels in responders and non-responders, respectively. 12_R/12_NR: expression levels after 12 months of teriflunomide treatment in responders and non-responders, respectively. DUSP6: dual specificity phosphatase 6. IFIT1: interferon induced protein with tetratricopeptide repeats 1. LRG1: leucine rich alpha-2-glycoprotein 1. MX1: MX dynamin like GTPase 1. SPARC: secreted protein acidic and cysteine rich. TNFRSF13C: TNF receptor superfamily member 13C. RSAD2: radical S-adenosyl methionine domain containing 2. ZBP1: Z-DNA binding protein 1. P-values were obtained after comparisons between groups by means of a Mann-Whitney U test. ns: p>0.05.


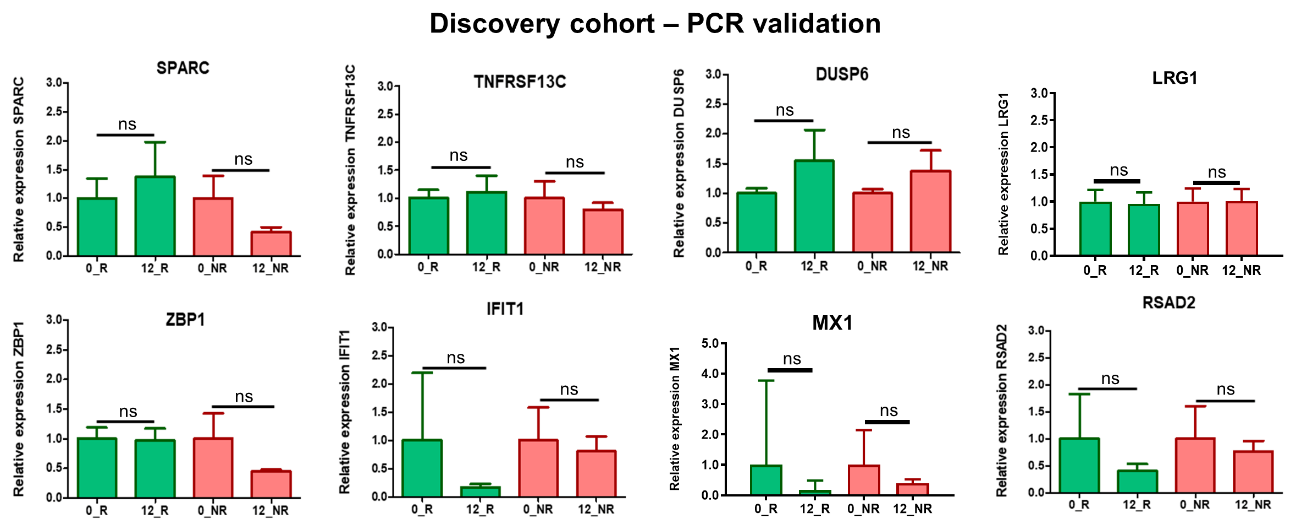


**Figure S3.** Expression levels of selected from the RNA-seq in the discovery cohort determined by real-time PCR at baseline. Bars and whiskers represent mean and standard error of the mean. 0_R/0_NR: baseline expression levels in responders and non-responders, respectively. 12_R/12_NR: expression levels after 12 months of teriflunomide treatment in responders and non-responders, respectively. DUSP6: dual specificity phosphatase 6. CLU: clusterin. EGR3: early growth response 3. IFI27: interferon alpha inducible protein 27. IFITM1: interferon induced transmembrane protein 1. IFIT1: interferon induced protein with tetratricopeptide repeats 1. LRG1: leucine rich alpha-2-glycoprotein 1. MX1: MX dynamin like GTPase 1. PF4: platelet factor 4. PPBP: pro-platelet basic protein. SPARC: secreted protein acidic and cysteine rich. TNFRSF13C: TNF receptor superfamily member 13C. RSAD2: radical S-adenosyl methionine domain containing 2. ZBP1: Z-DNA binding protein 1. P-values were obtained after comparisons between groups by means of a Mann-Whitney U test. *P<0.05, **p<0.01, ***p <0.001. ns: p>0.05.


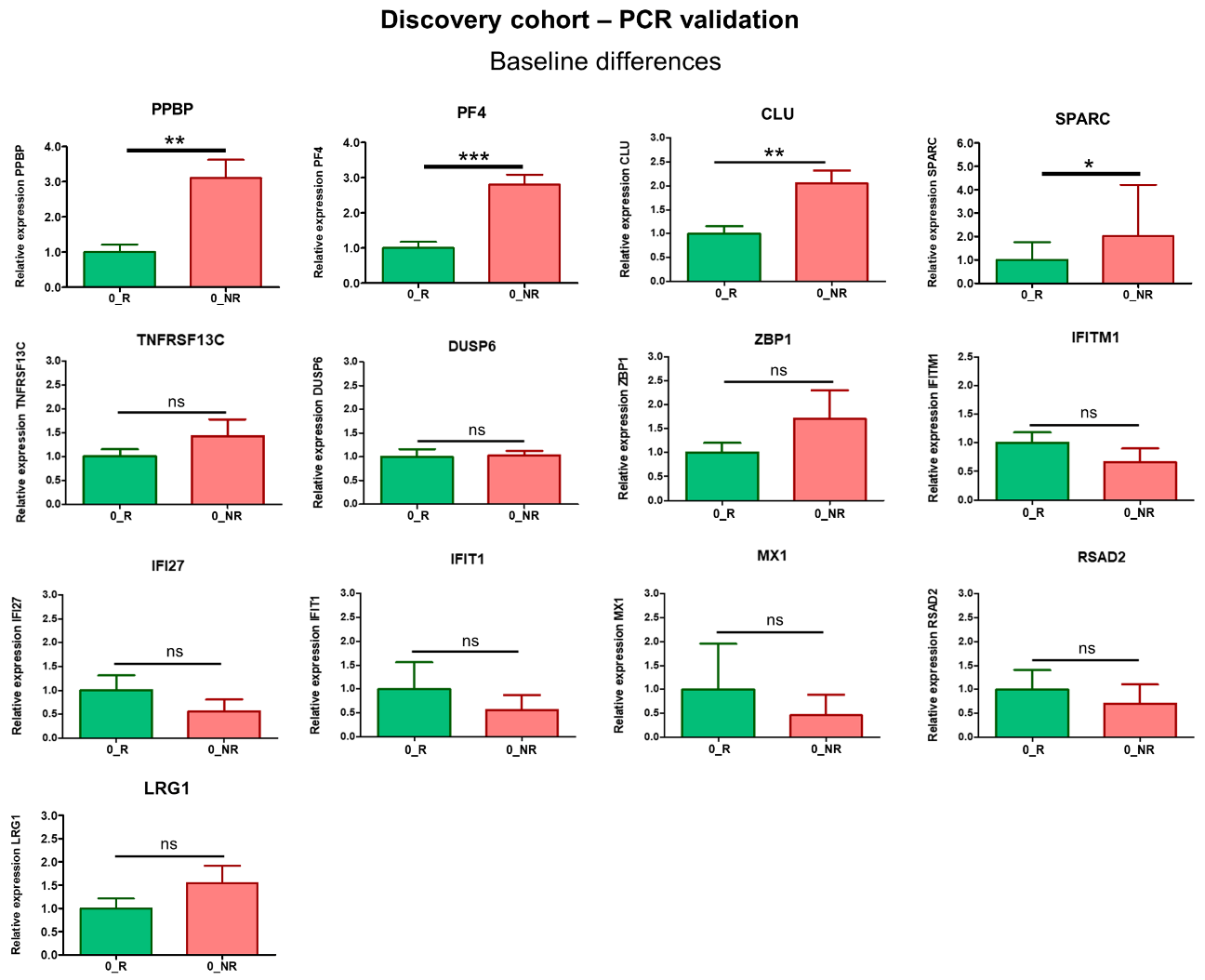


**Figure S4.** Expression levels of genes in PBMC from responders and non-responders to teriflunomide that were not validated by real-time PCR in the independent validation cohort. Bars and whiskers represent mean and standard error of the mean. 0_R/0_NR: baseline expression levels in responders and non-responders, respectively. 12_R/12_NR: expression levels after 12 months of teriflunomide treatment in responders and non-responders, respectively. PF4: platelet factor 4. PPBP: pro-platelet basic protein. IFI27: interferon alpha inducible protein 27. IFITM1: interferon induced transmembrane protein 1. P-values were obtained after comparisons between groups by means of a Mann-Whitney U test. ns: p>0.05.


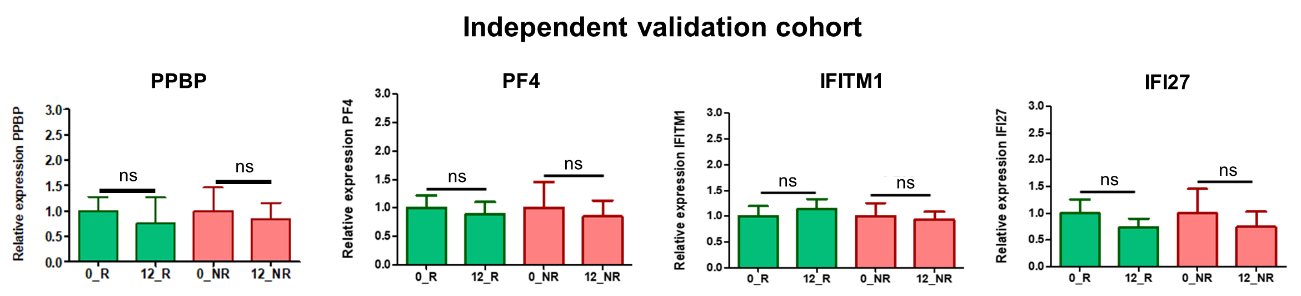


**Figure S5.** Baseline expression levels of genes determined by real-time PCR in the independent validation cohort. Bars and whiskers represent mean and standard error of the mean. 0_R/0_NR: baseline expression levels in responders and non-responders, respectively. CLU: clusterin. IFI27: interferon alpha inducible protein 27. IFITM1: interferon induced transmembrane protein 1. PF4: platelet factor 4. PPBP: pro-platelet basic protein. P-values were obtained after comparisons between groups by means of a Mann-Whitney U test. ns: p>0.05.


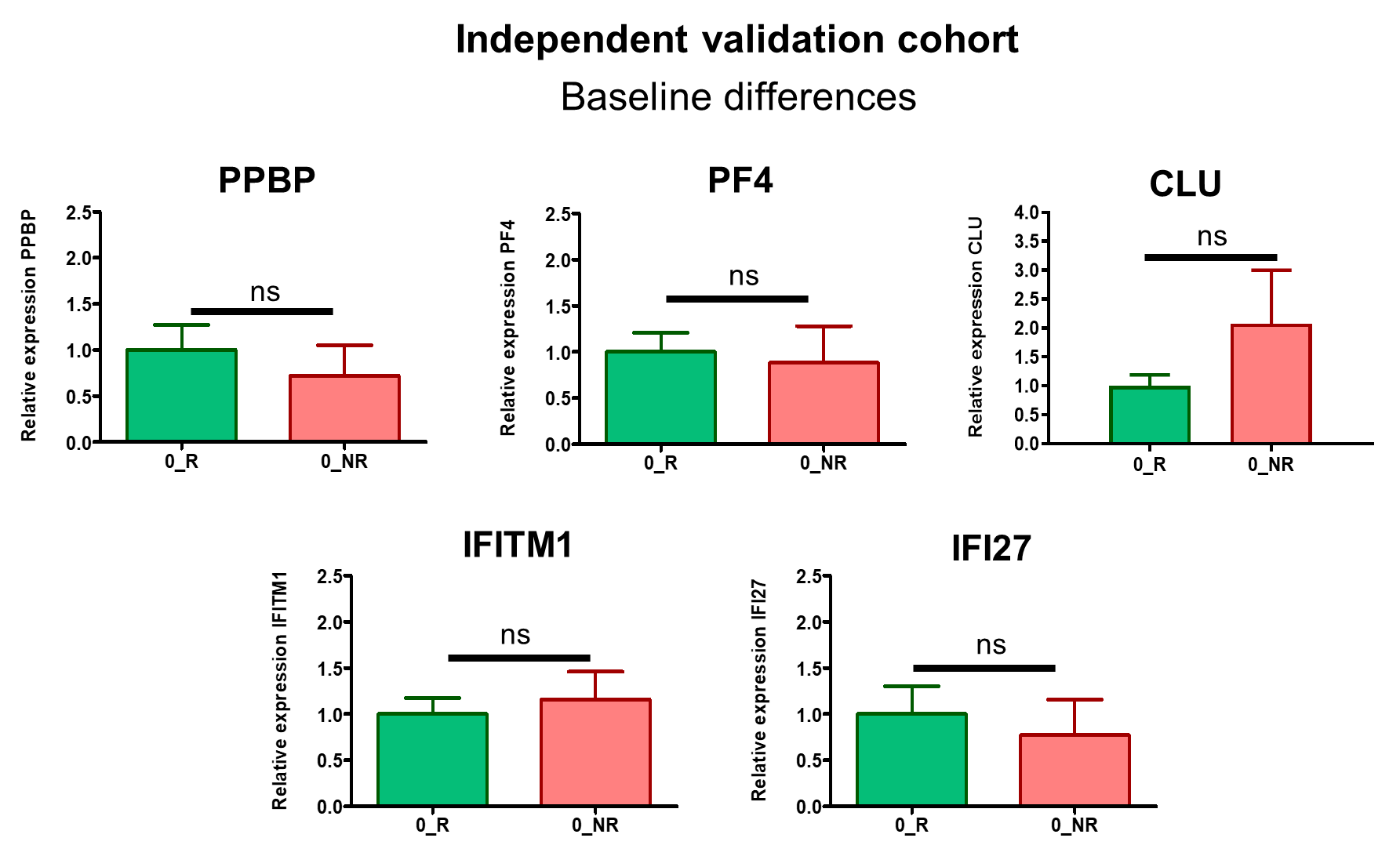


**Figure S6**. Subcellular fractions were obtained through differential centrifugations. The efficacy of the procedure was checked by studying the presence/absence of specific proteins for the mitochondrial and cytosolic fractions. (A) Mitochondrial and cytosolic fractions of samples incubated with the mitocondrial marker MnSOD (manganese superoxide dismutase). (B) Mitochondrial and cytosolic fractions of the samples incubated with the cytosolic marker caspase 3. Cyt: cytosolic. Mit: mitochondrial.


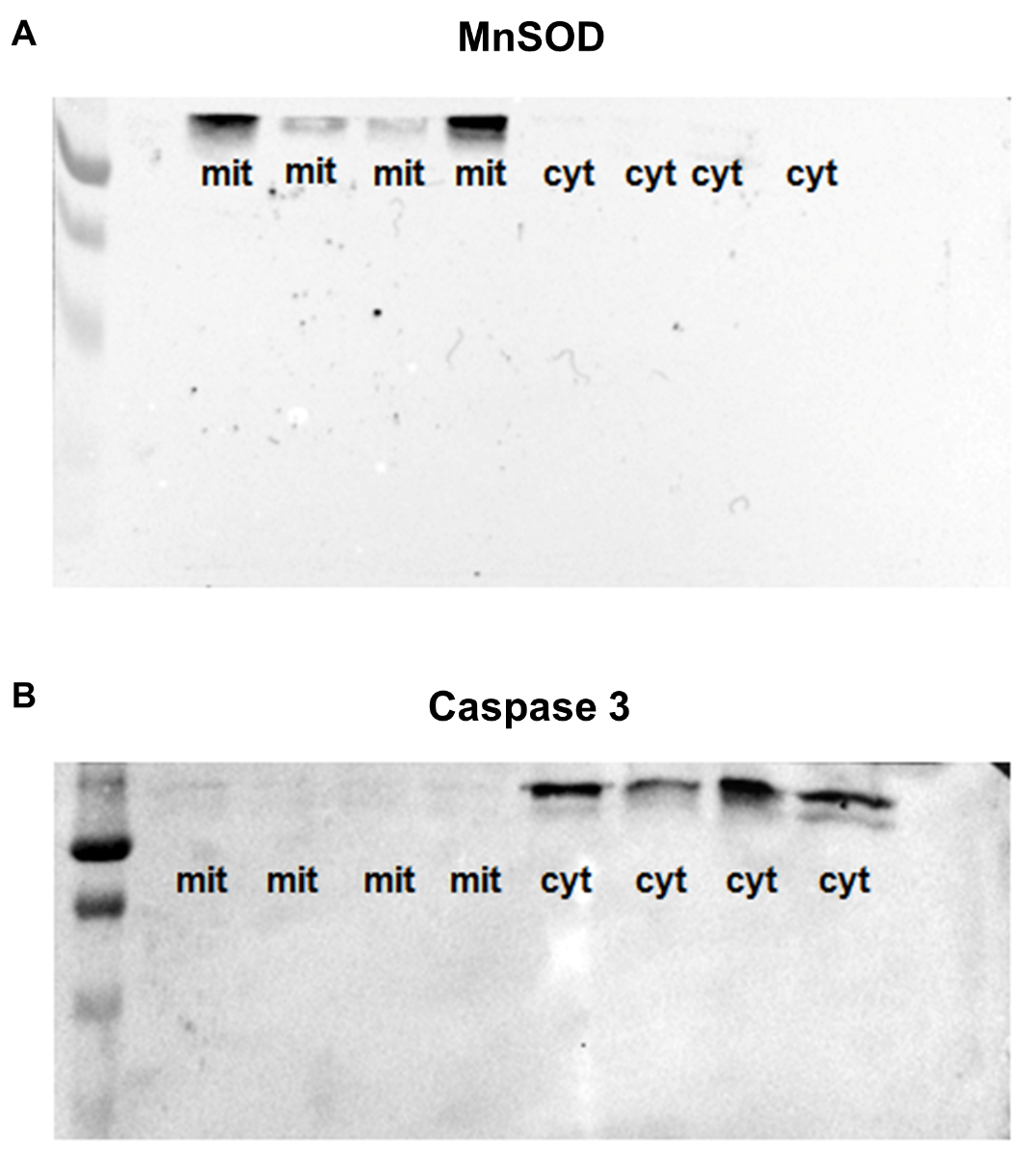


**Figure S7.** Membrane depolarization, superoxide content and mitochondrial mass upon PHA stimulation of PBMC (A) and T-lymphocytes (B) from responders (N=5) and non-responders (N=6) to teriflunomide. Mitochondrial depolarization was evaluated using MitoProbe™ TMRM in 7AAD and Annexin V negative cells. Detection of the mitochondrial superoxide was assessed with MitoSOX^TM^ Red. Mitochondrial mass was measured with Mitotracker Green^TM^ (ratio of the median fluorescence intensity in the presence/absence of PHA) by flow cytometry analysis. No significant differences were observed between whole PBMC or CD3^+^ T cells of responders and non-responders after 12 months of treatment. Bars and whiskers represent mean and standard deviation. 12_R/12_NR: levels of parameters after 12 months of teriflunomide treatment in responders and non-responders, respectively. PBMC: peripheral blood mononuclear cells.

**
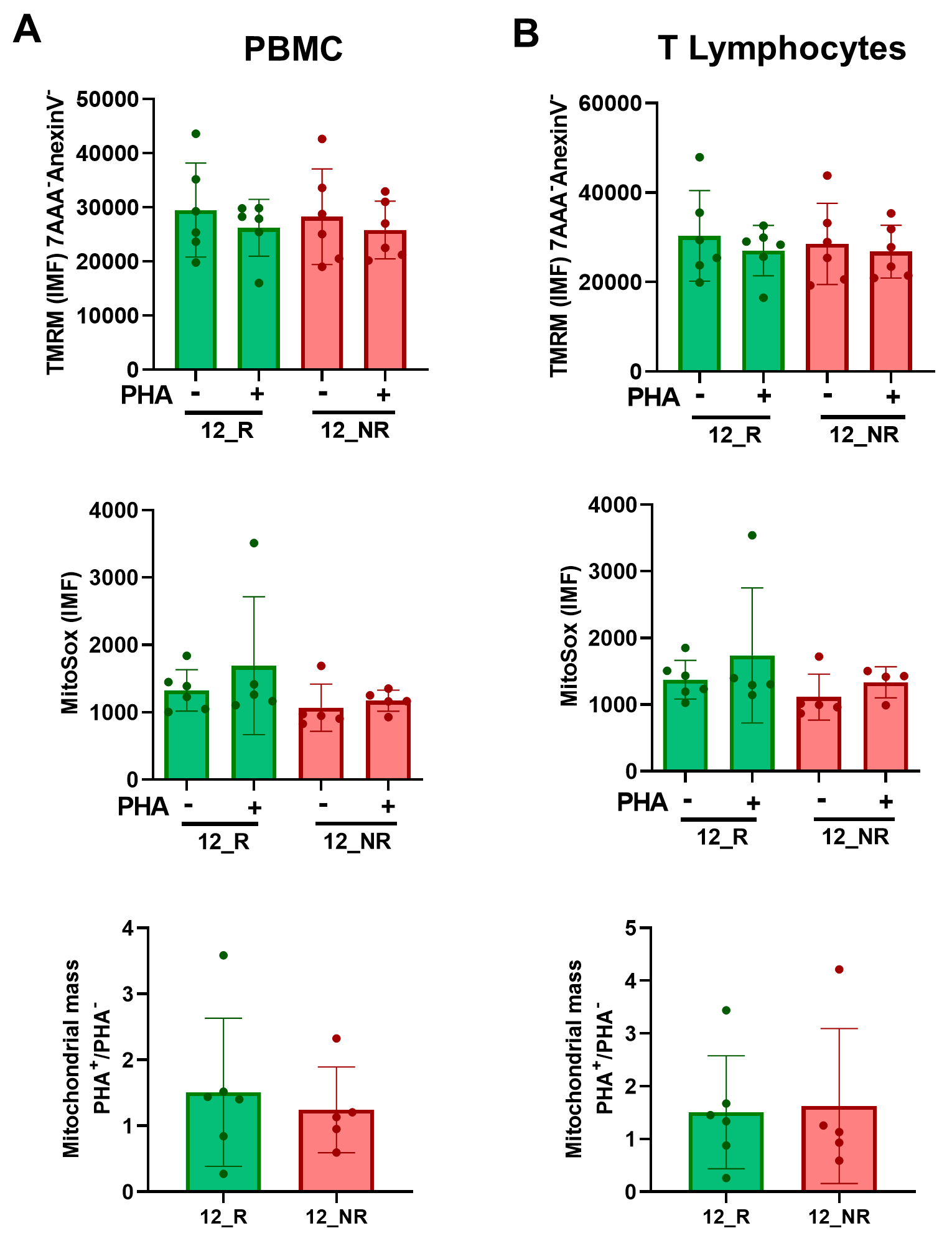
**

**Figure S8.** Gating strategies to analyze cell proliferation (A,B) and apoptosis (C,D) in PBMC from teriflunomide responders and non-responders. (A) Lymphocytes were defined by SSC-Area and FSC-Area. FSC-Height vs FSC-Area were used to exclude cell aggregates from the analysis. Dead cells were excluded with a live/dead marker. Total T cells were selected gating on CD3^+^ T cells. T cells were further divided into CD4^+^/CD8^-^ and CD8^+^/CD4^-^ subsets using a CD4 vs. CD8 plot, and T cell phenotypes were defined based on different combinations of CCR7 and CD45RA, naive T (TN) cells (CCR7^+^/CD45RA^+^), effector memory T (TEM) cells (CCR7^−^/CD45RA^−^), central memory T (TCM) cells (CCR7^+^/CD45RA^−^), and terminally differentiated T (TE) cells (CCR7^−^/CD45RA^+^). (B) A representative scheme of proliferating cells based on CFSE decay is shown. (C) Lymphocytes were gated on the basis of their FSC-A and SSC-Area. Doublets were excluded by the FSC-Height vs FSC-Area. T cells were selected by the positive staining for CD3 and were further divided into CD4^+^/CD8^-^ and CD8^+^/CD4^-^ subsets using a CD4 vs CD8 plot. T cell phenotypes were defined based on different combinations of CCR7 and CD45RA, naive T (TN) cells (CCR7^+^/CD45RA^+^), effector memory T (TEM) cells (CCR7^−^/CD45RA^−^), central memory T (TCM) cells (CCR7^+^/CD45RA^−^), and terminally differentiated T (TE) cells (CCR7^−^/CD45RA^+^). (B) A representative panel to quantify apoptosis based on Annexin V/7AAD staining is depicted.

**
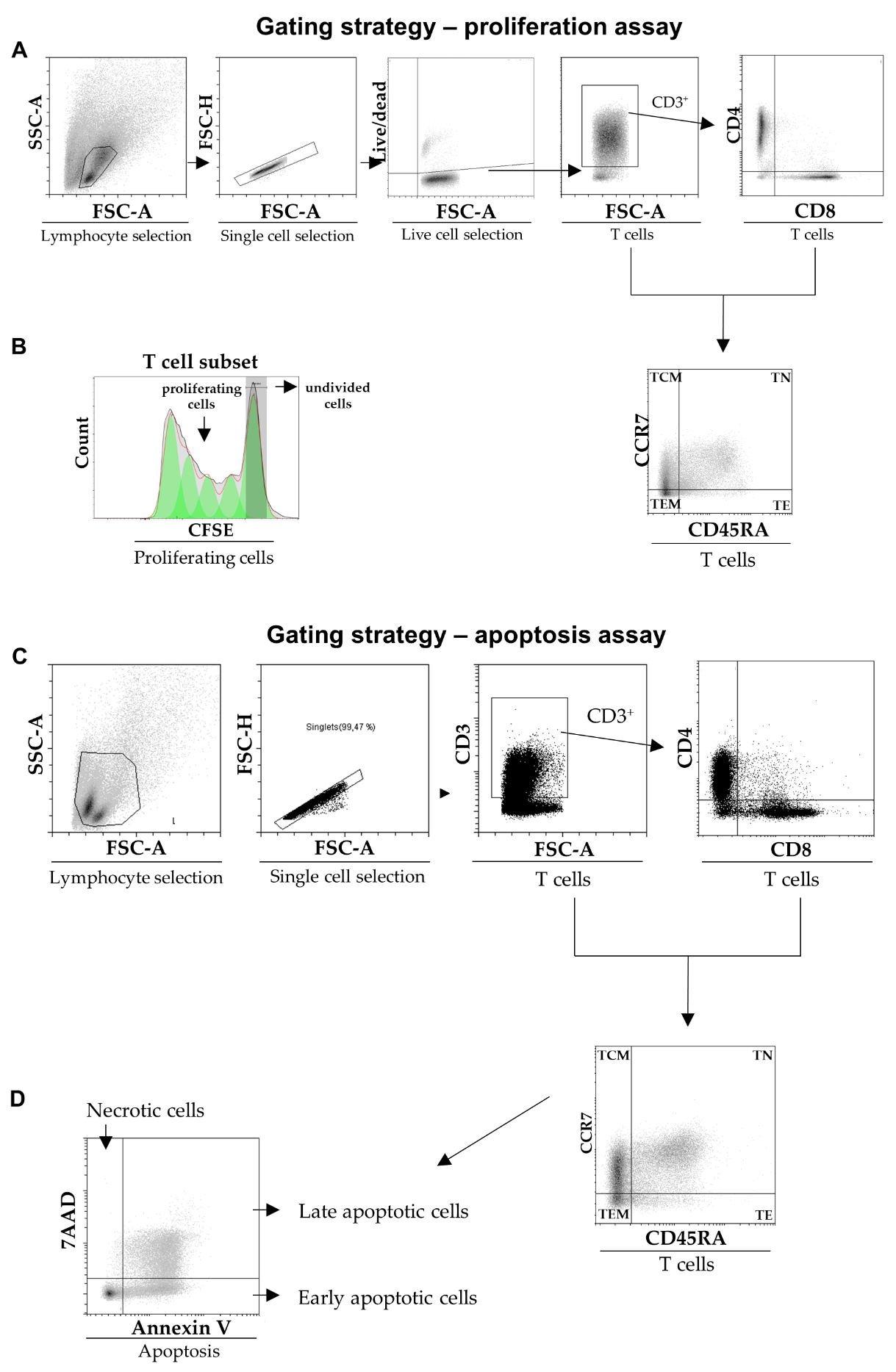
**

**Figure S9.** Bars showing late apoptosis/necrosis of T cells after stimulation with staurosporine at 2µM for 16 hours. Apoptosis was determined by means of Annexin V/7AAD staining using the FlowJo software in CD3^+^, CD3^+^CD4^+^, and CD3^+^CD8^+^ T cells. Percentages of gated CD3^+^, CD3^+^CD4^+^ and CD3^+^CD8^+^ that show positivity for Annexin V and 7AAD (late apoptotic/necrosis) are represented. No significant differences were observed across groups. Results are given as mean and standard error of the mean. 0 and 12 indicate percentages at baseline and 12 months of treatment respectively in responders (R) and non-responders (NR).


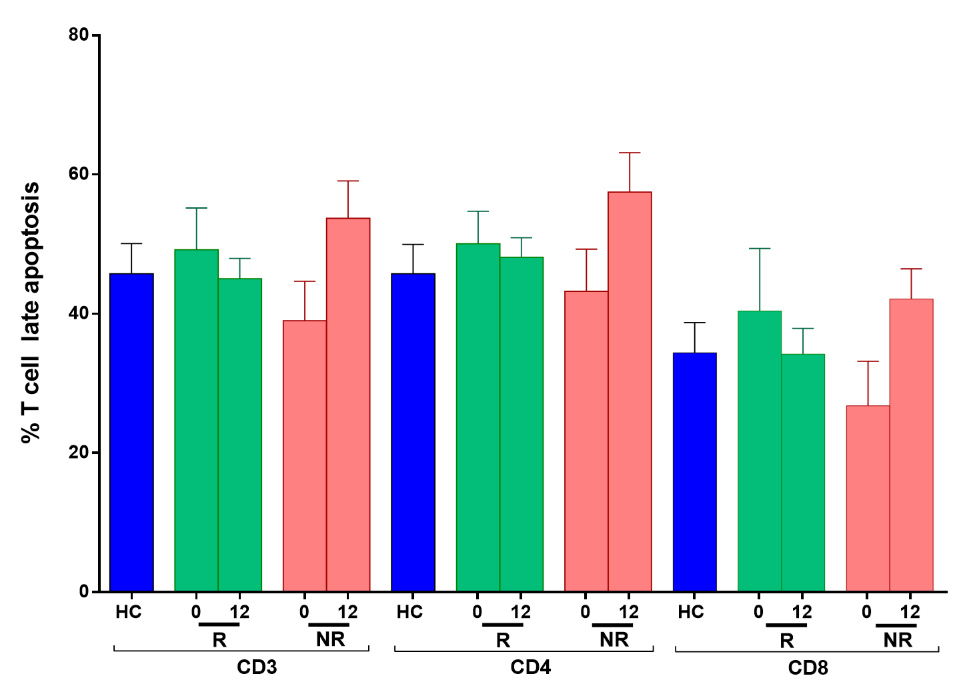

Supplement: Supplementary file 1 — Supporting Information [file CTM2-14-e1654-s004.docx]
