# Supplementary material for: Clusterin deficiency is associated with a lack of response to teriflunomide in multiple sclerosis
Source: Clin Transl Med. 2024 Apr 9;14(4):e1654. doi: 10.1002/ctm2.1654 (PMC11003271; doi:10.1002/ctm2.1654)
Supplement: Supplementary file 2 — Supporting Information [file CTM2-14-e1654-s008.doc]

**Table S1**. Demographic and clinical characteristics of MS patients included in the discovery cohort for RNA-seq.

| Characteristics | Responders | Non-responders | P value |
| --- | --- | --- | --- |
| N | 11 | 10 | - |
| Female/male (% women) | 8/3 (72.7) | 8/2 (80.0) | 0.696 |
| Age (years) | 45.8 (4.5) | 41.8 (10.3) | 0.257 |
| Duration of disease (years) | 10.5 (5.4) | 10.9 (8.7) | 0.916 |
| EDSS^a^ | 1.5 (1.0 - 2.0) | 1.5 (1.4 - 2.8) | 0.663 |
| Number of T2 lesions^b,c^ [n (%)] |  |  |  |
| 1 | 3 (27.3) | 0 (0) | 0.203 |
| 2 | 3 (27.3) | 4 (40.0) |  |
| 3 | 5 (45.5) | 6 (60.0) |  |
| Nº of Gd-enhancing lesions^b^ | 0.2 (0.4) | 0.6 (1.3) | 0.311 |
| Naïve / no naïve (% naïve)^d^ | 4/7 (36.4) | 3/7 (30.0) | 0.757 |

Age, disease duration, and EDSS are calculated in relation to the date of teriflunomide treatment onset. Data are expressed as mean (standard deviation) unless otherwise stated. ^a^Data are expressed as median (interquartile range). ^b^Refers to the number of T2 and gadolinium (Gd)-enhancing lesions at baseline MRI. ^c^T2 lesions were classified in the following 3 categories according to the number of lesions: 1 = 1–3 lesions, 2 = 4–9 lesions, 3 = ≥10 lesions. ^d^Before teriflunomide, in the responder group 7 patients were treated with platform therapies (interferon-beta or glatiramer acetate) and one of these patients was also treated before with dimethyl fumarate; in the non-responder group 7 patients were treated with platform therapies. P-values were obtained after comparisons between responders and non-responders by means of the χ2 test (sex, % naïve patients, and number of T2 lesions), Mann-Whitney U test (EDSS), and Student t test (remaining variables). EDSS: Expanded Disability Status Scale.

**Table S2.** Remaining genes showing differential expression in non-responders but not in responders with p-values >0.001 between the baseline and 12 months of teriflunomide treatment obtained in the discovery cohort by RNA-seq.

| Gene | Description | 0 vs. 12 Responders  FC p-value adj p-value | | | | | 0 vs. 12 Non-responders  FC p-value adj p-value | | | | |  |
| --- | --- | --- | --- | --- | --- | --- | --- | --- | --- | --- | --- | --- |
| **PAX5** | paired box 5 | -1.3 | | 0.064 | 1.000 | | -1.7 | 3.3 x 10^-6^ | | 0.002 | |  |
| **CAVIN2** | caveolae associated protein 2 | 1.5 | | 0.224 | 1.000 | | -1.9 | 4.6 x 10^-6^ | | 0.003 | |  |
| **GNAZ** | G protein subunit alpha z | 1.0 | | 0.830 | 1.000 | | -2.1 | 7.3 x 10^-6^ | | 0.005 | |  |
| **FADS3** | fatty acid desaturase 3 | -1.3 | | 0.115 | 1.000 | | -1.6 | 1.0 x 10^-5^ | | 0.006 | |  |
| **ITGB3** | integrin subunit beta 3 | 1.4 | | 0.165 | 1.000 | | -2.2 | 1.2 x 10^-5^ | | 0.007 | |  |
| **AQP10** | aquaporin 10 | -1.9 | | 0.170 | 1.000 | | -3.6 | 1.5 x 10^-5^ | | 0.008 | |  |
| **LTBP1** | latent transforming growth factor beta binding protein 1 | 1.0 | | 0.879 | 1.000 | | -2.3 | 1.7 x 10^-5^ | | 0.008 | |  |
| **CTDSPL** | CTD small phosphatase like | -1.1 | | 0.751 | 1.000 | | -2.1 | 2.1 x 10^-5^ | | 0.010 | |  |
| **SEPTIN5** | septin 5 | 1.2 | | 0.471 | 1.000 | | -2.1 | 2.2 x 10^-5^ | | 0.010 | |  |
| **EGR1** | early growth response 1 | -1.4 | | 0.631 | 1.000 | | -6.0 | 2.6 x 10^-5^ | | 0.010 | |  |
| **TREML1** | triggering receptor expressed on myeloid cells like 1 | 1.3 | | 0.285 | 1.000 | | -2.0 | 2.7 x 10^-5^ | | 0.011 | |  |
| **GP9** | glycoprotein IX platelet | -1.1 | | 0.779 | 1.000 | | -2.0 | 2.8 x 10^-5^ | | 0.011 | |  |
| **CD22** | CD22 molecule | -1.3 | | 0.061 | 1.000 | | -1.6 | 3.1 x 10^-5^ | | 0.011 | |  |
| Gene | Description | 0 vs. 12 Responders  FC p-value adj p-value | | | | | 0 vs. 12 Non-responders  FC p-value adj p-value | | | | |  |
| **PRKAR2B** | protein kinase cAMP-dependent type II regulatory subunit beta | 1.3 | 0.125 | | | 1.000 | -1.6 | | 3.2 x 10^-5^ | | 0.011 |  |
| **P2RX5** | purinergic receptor P2X 5 | -1.2 | 0.155 | | | 1.000 | -1.4 | | 3.2 x 10^-5^ | | 0.011 |  |
| **ITGA2B** | integrin subunit alpha 2b | 1.1 | 0.539 | | | 1.000 | -2.9 | | 3.3 x 10^-5^ | | 0.011 |  |
| **CCR2** | C-C motif chemokine receptor 2 | 1.6 | 0.154 | | | 1.000 | 1.4 | | 4.0 x 10^-5^ | | 0.013 |  |
| **HBB** | hemoglobin subunit beta | 2.6 | 0.138 | | | 1.000 | 11.8 | | 4.4 x 10^-5^ | | 0.014 |  |
| **HOOK2** | hook microtubule tethering protein 2 | -1.3 | 0.310 | | | 1.000 | -1.7 | | 4.9 x 10^-5^ | | 0.014 |  |
| **FAM111B** | family with sequence similarity 111 member B | -1.5 | 0.112 | | | 1.000 | -2.4 | | 5.6 x 10^-5^ | | 0.016 |  |
| **POU2AF1** | POU class 2 homeobox associating factor 1 | -1.2 | 0.178 | | | 1.000 | -1.5 | | 5.7 x 10^-5^ | | 0.016 |  |
| **TUBB1** | tubulin beta 1 class VI | 1.5 | 0.281 | | | 1.000 | -1.7 | | 7.2 x 10^-5^ | | 0.019 |  |
| **CMTM5** | CKLF like MARVEL transmembrane domain containing 5 | 1.2 | 0.534 | | | 1.000 | -1.9 | | 7.5 x 10^-5^ | | 0.019 |  |
| **EGR2** | early growth response 2 | -1.3 | 0.663 | | | 1.000 | -4.9 | | 1.0 x 10^-4^ | | 0.025 |  |
| **MAFF** | MAF bZIP transcription factor F | -1.8 | 0.182 | | | 1.000 | -1.8 | | 1.1 x 10^-4^ | | 0.025 |  |
| **AFF3** | AF4/FMR2 family member 3 | -1.2 | 0.116 | | | 1.000 | -1.4 | | 1.2 x 10^-4^ | | 0.028 |  |
| **TSPAN33** | tetraspanin 33 | -1.0 | 0.851 | | | 1.000 | -1.5 | | 1.3 x 10^-4^ | | 0.029 |  |
| **IER2** | immediate early response 2 | -1.1 | 0.766 | | | 1.000 | -1.6 | | 1.4 x 10^-4^ | | 0.030 |  |
| Gene | Description | 0 vs. 12 Responders  FC p-value adj p-value | | | | | 0 vs. 12 Non-responders  FC p-value adj p-value | | | | |  |
| **PGRMC1** | progesterone receptor membrane component 1 | 1.1 | 0.413 | | | 1.000 | -1.5 | | 1.4 x 10^-4^ | | 0.030 |  |
| **ABLIM3** | actin binding LIM protein family member 3 | -1.0 | 0.864 | | | 1.000 | -2.1 | | 1.6 x 10^-4^ | | 0.032 |  |
| **NLRC4** | NLR family CARD domain containing 4 | 1.3 | 0.157 | | | 1.000 | 1.5 | | 2.0 x 10^-4^ | | 0.038 |  |
| **ESAM** | endothelial cell adhesion molecule | 1.0 | 0.858 | | | 1.000 | -1.7 | | 2.1 x 10^-4^ | | 0.040 |  |
| **LGALS3BP** | galectin 3 binding protein | -1.9 | 0.052 | | | 1.000 | -1.6 | | 2.1 x 10^-4^ | | 0.040 |  |
| **BEND2** | BEN domain containing 2 | 1.1 | 0.672 | | | 1.000 | -2.0 | | 2.5 x 10^-4^ | | 0.047 |  |
| **GNG11** | G protein subunit gamma 11 | 1.5 | 0.098 | | | 1.000 | -1.7 | | 2.6 x 10^-4^ | | 0.048 |  |
| **COL19A1** | collagen type XIX alpha 1 chain | -1.2 | 0.133 | | | 1.000 | -1.5 | | 2.7 x 10^-4^ | | 0.048 |  |
| **LGALSL** | galectin like | 1.2 | 0.465 | | | 1.000 | -1.9 | | 2.7 x 10^-4^ | | 0.049 |  |

Positive fold changes (FC) indicate up-regulated between the baseline and the 12 months treated time point, whereas negative FC denote down-regulated genes between the baseline and 12 months treatment. Adj p-value: FDR (false discovery rate)-adjusted p values (<0.05).

**Table S3**. Demographic and clinical characteristics of MS patients included in the validation cohort for real time PCR.

| Characteristics | Responders | Non-responders | P value |
| --- | --- | --- | --- |
| N | 11 | 10 | - |
| Female/male (% women) | 6/5 (54.5) | 4/6 (40.0) | 0.505 |
| Age (years) | 42.0 (8.2) | 40.5 (5.3) | 0.616 |
| Duration of disease (years) | 7.1 (6.6) | 7.8 (5.7) | 0.793 |
| EDSS^a^ | 2.0 (2.0 - 3.5) | 1.5 (1.5 - 3.4) | 0.314 |
| Number of T2 lesions^b,c^ [n (%)] |  |  |  |
| 1 | 0 (0) | 0 (0) | 0.314 |
| 2 | 3 (27.3) | 1 (10.0) |  |
| 3 | 8 (72.7) | 9 (90.0) |  |
| Nº of Gd-enhancing lesions^b^ | 0.1 (0.3) | 0.6 (1.3) | 0.210 |
| Naïve / no naïve (% naïve)^d^ | 4/7 (36.4) | 3/7 (30.0) | 0.757 |

Age, disease duration and EDSS are calculated in relation to the date of teriflunomide treatment onset. Data are expressed as mean (standard deviation) unless otherwise stated. ^a^Data are expressed as median (interquartile range). ^b^Refers to the number of T2 and gadolinium (Gd)-enhancing lesions at baseline MRI. ^c^T2 lesions were classified in the following 3 categories according to the number of lesions: 1 = 1–3 lesions, 2 = 4–9 lesions, 3 = ≥10 lesions. ^d^Before teriflunomide, in the responder group 6 patients were treated with platform therapies (interferon-beta or glatiramer acetate) and one patient received previously platform therapies, dimethyl fumarate, and natalizumab; in the non-responder group 6 patients were treated with platform therapies and one patient received before fingolimod and natalizumab. P-values were obtained after comparisons between responders and non-responders by means of the χ2 test (sex, % naïve patients, and number of T2 lesions), Mann-Whitney U test (EDSS), and Student t test (remaining variables). EDSS: Expanded Disability Status Scale.

**Table S4**. Demographic and clinical characteristics of MS patients treated with dimethyl fumarate and fingolimod.

| Characteristics | Responders | Dimethyl fumarate  Non-responders | P value |  | Responders | Fingolimod  Non-responders | P value |
| --- | --- | --- | --- | --- | --- | --- | --- |
| N | 7 | 5 | - |  | 4 | 6 | - |
| Female/male (% women) | 5/2 (71.4) | 4/1 (80.0) | 0.762 |  | 2/2 (50.0) | 5/1 (83.3) | 0.312 |
| Age (years) | 40.0 (9.6) | 42.0 (7.4) | 0.706 |  | 45.5 (7.2) | 41.4 (8.7) | 0.471 |
| Duration of disease (years) | 7.6 (8.2) | 4.7 (3.4) | 0.481 |  | 12.9 (4.0) | 13.6 (10.9) | 0.902 |
| EDSS^a^ | 2.2 (1.0 - 3.0) | 1.6 (1.2 - 2.0) | 0.272 |  | 4.0 (1.6 - 6.8) | 3.0 (1.8 - 4.1) | 0.492 |
| Number of T2 lesions^b,c^ [n (%)] |  |  |  |  |  |  |  |
| 1 | 0 (0) | 0 (0) | 0.228 |  | 0 (0) | 0 (0) | 0.333 |
| 2 | 1 (14.2) | 0 (0) |  |  | 0 (0) | 0 (0) |  |
| 3 | 6 (85.8) | 5 (100.0) |  |  | 4 (100.0) | 6 (100.0) |  |
| Nº of Gd-enhancing lesions^c^ | 0.3 (0.7) | 1.0 (2.2) | 0.444 |  | 0.0 (0.0) | 0.17 (0.41) | 0.447 |
| Naïve / no naïve (% naïve) | 2/5 (28.5) | 2/3 (40.0) | 0.815 |  | 0/4 (0.0) | 0/6 (0.0) | 1.000 |

Age, disease duration, and EDSS are calculated in relation to the date of treatment onset. Data are expressed as mean (standard deviation) unless otherwise stated. ^a^Data are expressed as median (interquartile range). ^b^Refers to the number of T2 and gadolinium (Gd)-enhancing lesions at baseline MRI. ^c^T2 lesions were classified in the following 3 categories according to the number of lesions: 1 = 1–3 lesions, 2 = 4–9 lesions, 3 = ≥10 lesions. P-values were obtained after comparisons between responders and non-responders by means of the χ2 test (sex, % naïve patients, and number of T2 lesions), Mann-Whitney U test (EDSS), and Student t test (remaining variables). EDSS: Expanded Disability Status Scale.

**Table S5**. Monoclonal antibodies used for peripheral blood cell immunophenotyping.

| **Monoclonal Antibody** | **Fluorochrome Conjugate** | **Manufacturer** |
| --- | --- | --- |
| Clusterin | FITC | Abcam PLC |
| CCR7 | PE | BD Biosciences |
| CD3 |
| CD19 |
| CD56 |
| CD11 | PE-Cy5 |
| CD56 | PerCP-Cy5.5 |
| CD19 | PE-Cy7 |
| CD45RO | APC |
| CD123 |
| CD8 | APC-H7 |
| CD14 |
| CD3 | BV-421 |
| HLA-DR | V-450 |
| CD45 | V-500 |

**Table S6.**

**Antibody panel used for staining of PBMC in** the apoptosis assay.

| **Target** | **Clone** | **Fluorochrome** | **Catalog** | **Vendor** | **Purpose** |
| --- | --- | --- | --- | --- | --- |
| CD3 | UCHT1 | FITC | 555332 | BD | Lineage |
| CD4 | SK3 | Alexa Fluor 700 | 566318 | BD |
| CD8 | SK1 | BV510 | 563919 | BD |
| CCR7 | G043H7 | PE | 353204 | Bio |
| CD45RA | Clone 5H9 | BV605 | 740424 | BD |
| 7AAD |  |  | 559925 | BD | Necrosis marker |
| Annexin V | R19-760 | APC | 550474 | BD | Apoptotic marker |

BD = BD Biosciences, Bio = BioLegend

**Antibody panel used for staining of PBMC in** the CFSE-proliferation assay.

| **Target** | **Clone** | **Fluorochrome** | **Catalog** | **Vendor** | **Purpose** |
| --- | --- | --- | --- | --- | --- |
| CD3 | HIT3a | APC | 555342 | BD | Lineage |
| CD4 | SK3 | Alexa Fluor 700 | 566318 | BD |
| CD8 | SK1 | BV510 | 563919 | BD |
| CCR7 | G043H7 | PE | 353204 | Bio |
| CD45RA | Clone 5H9 | BV605 | 740424 | BD |
| CFSE |  |  | C34554 | TF | Proliferation marker |
| Fixable Viability Dye |  | eF450 | 65-0863-14 | eBio | Live / Dead discrimination |

BD = BD Biosciences, Bio = BioLegend, eBio = eBioscience, TF = ThermoFisher
