# Supplementary figures and images for: Clusterin deficiency is associated with a lack of response to teriflunomide in multiple sclerosis
Source: Clin Transl Med. 2024 Apr 9;14(4):e1654. doi: 10.1002/ctm2.1654 (PMC11003271; doi:10.1002/ctm2.1654)

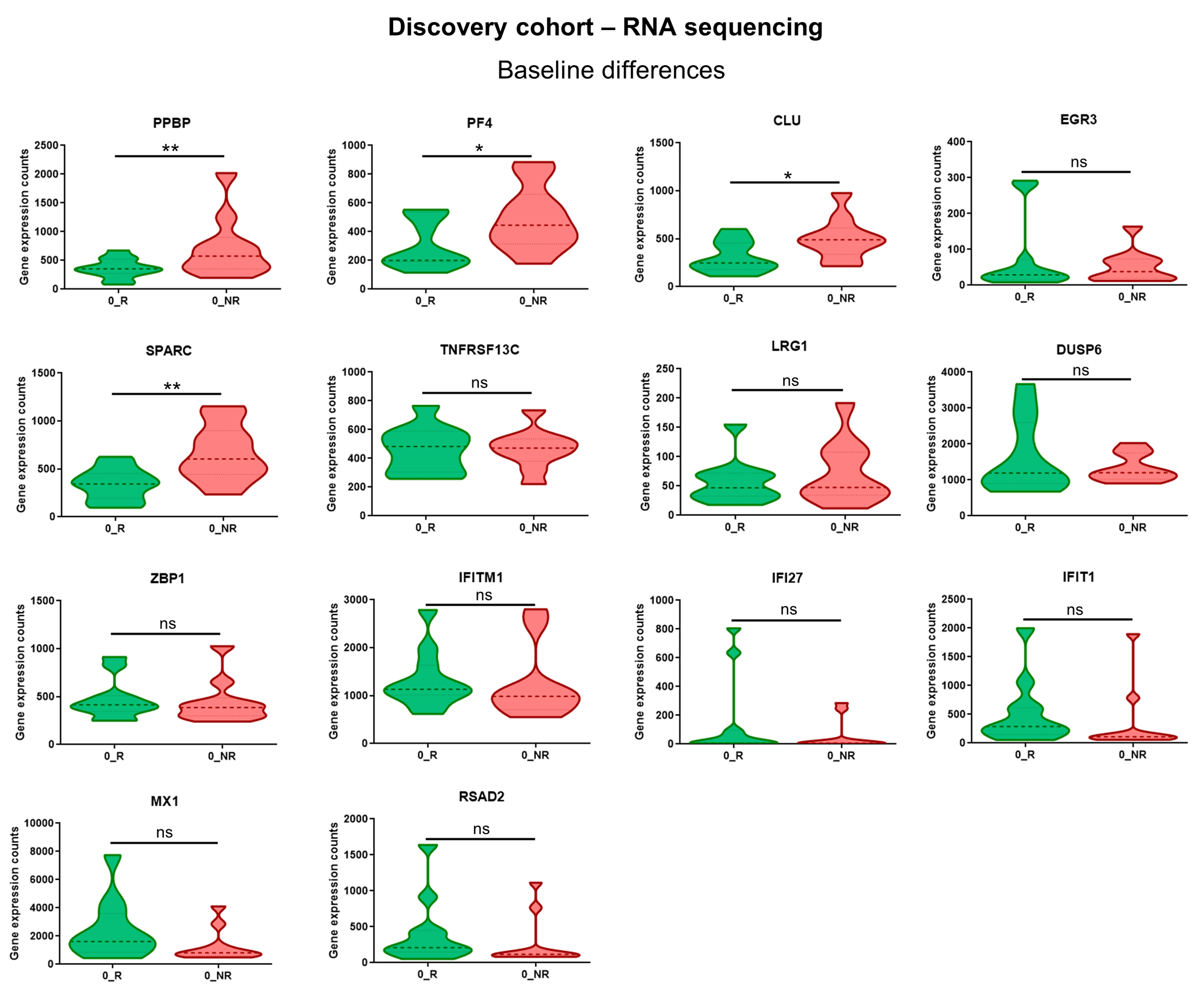

Supplement: Supplementary file 3 — Supporting Information [file CTM2-14-e1654-s001.tif]

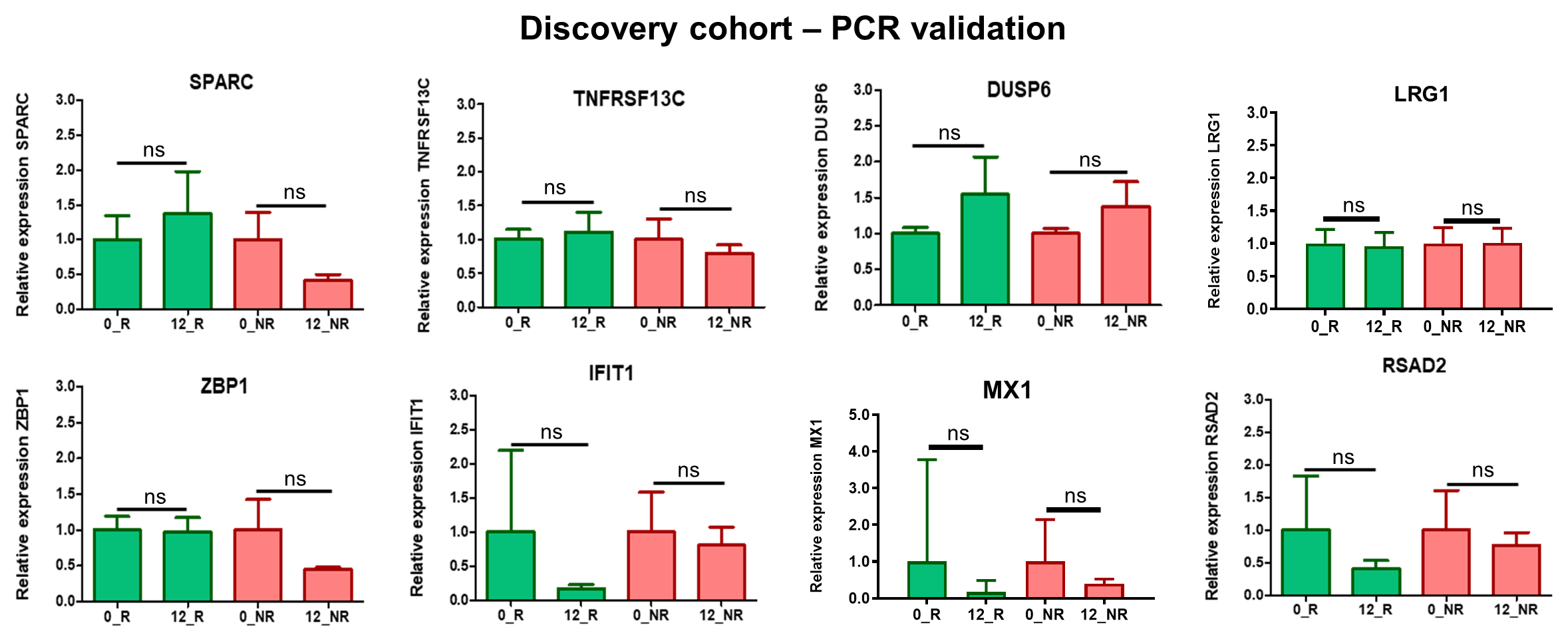

Supplement: Supplementary file 4 — Supporting Information [file CTM2-14-e1654-s003.tif]

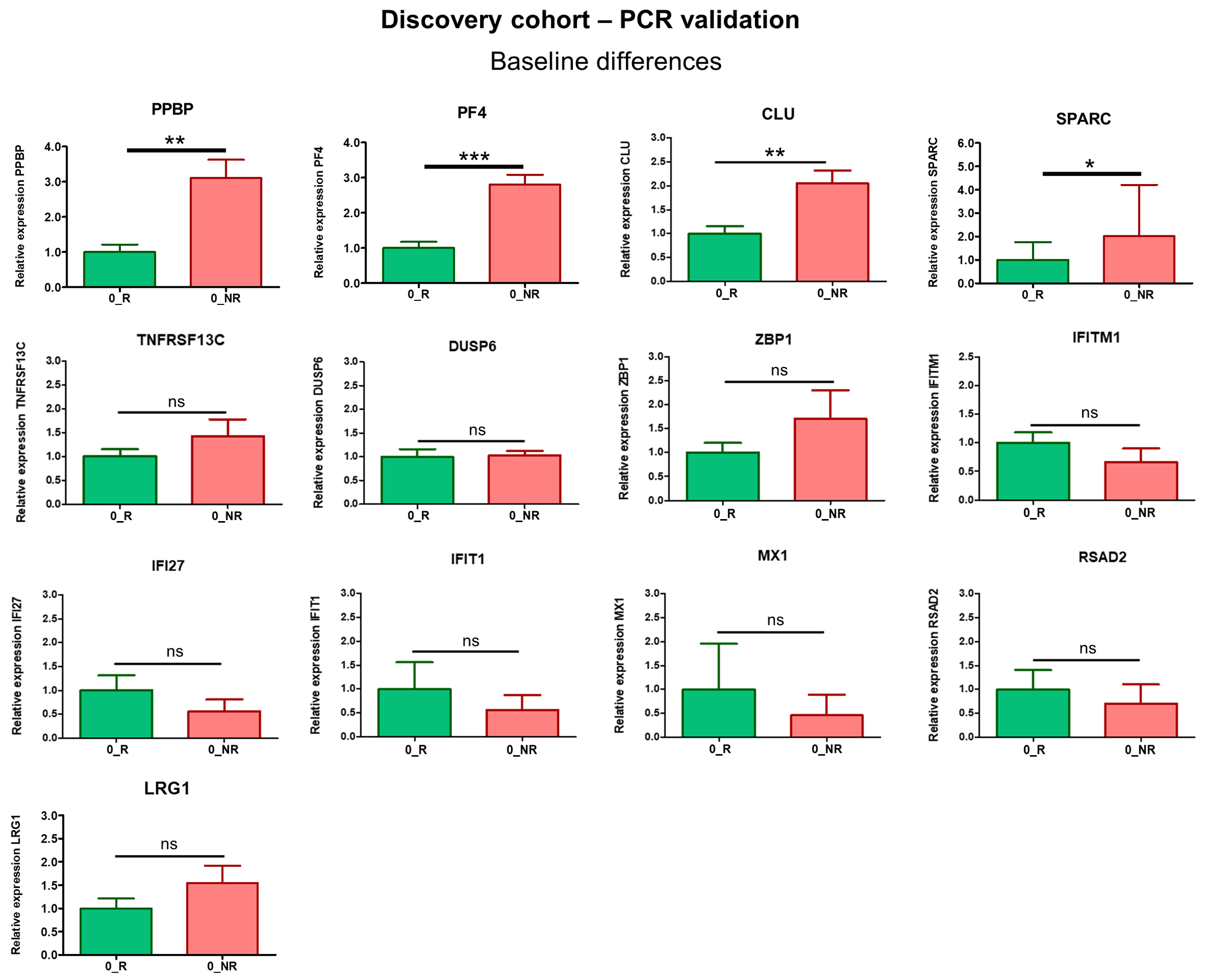

Supplement: Supplementary file 5 — Supporting Information [file CTM2-14-e1654-s005.tif]

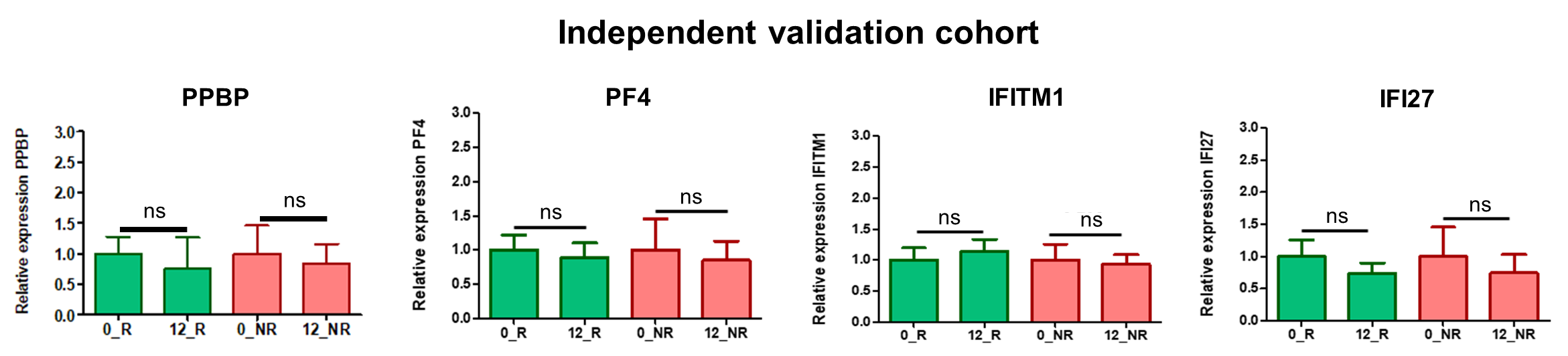

Supplement: Supplementary file 6 — Supporting Information [file CTM2-14-e1654-s009.tif]

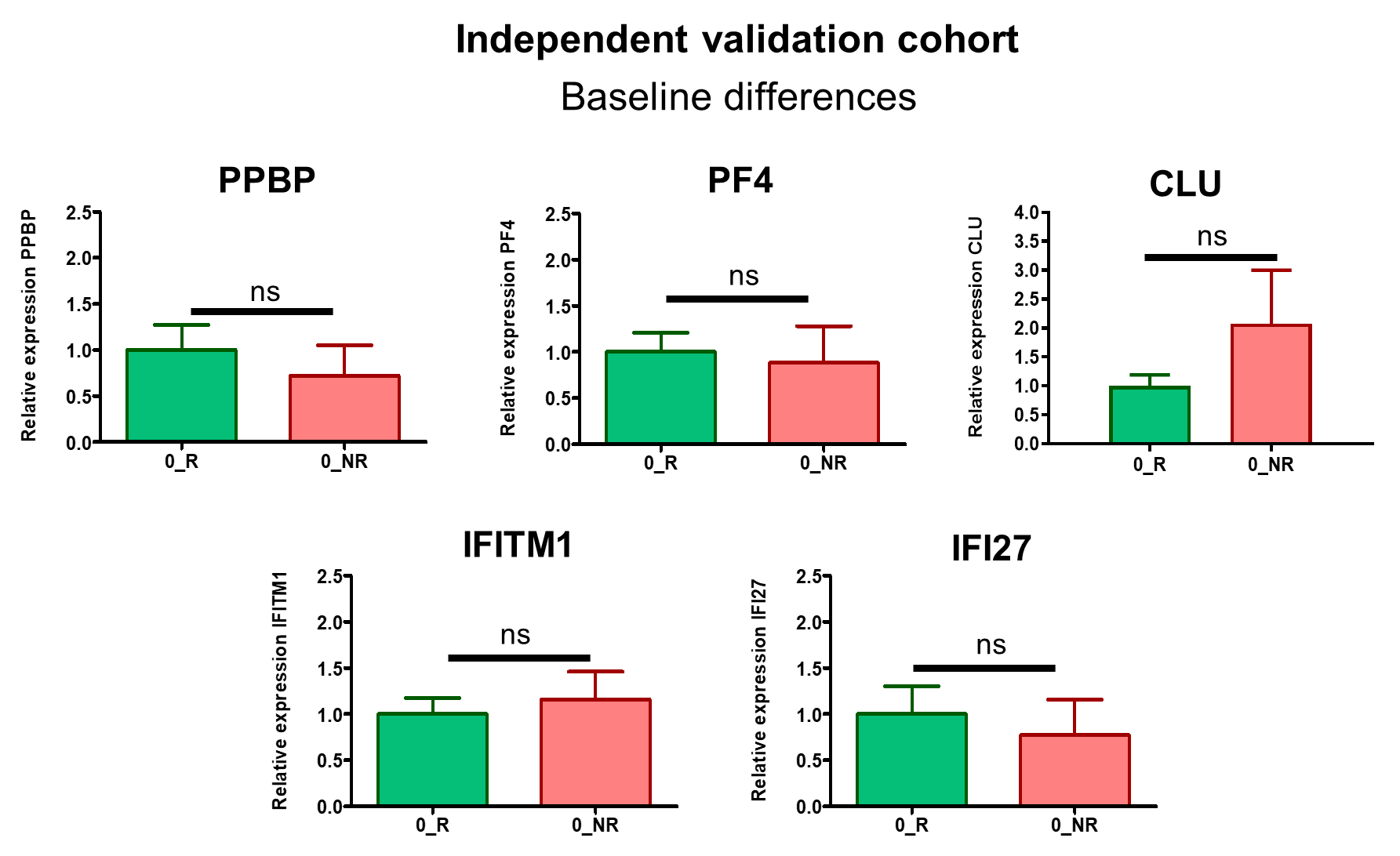

Supplement: Supplementary file 7 — Supporting Information [file CTM2-14-e1654-s007.tif]

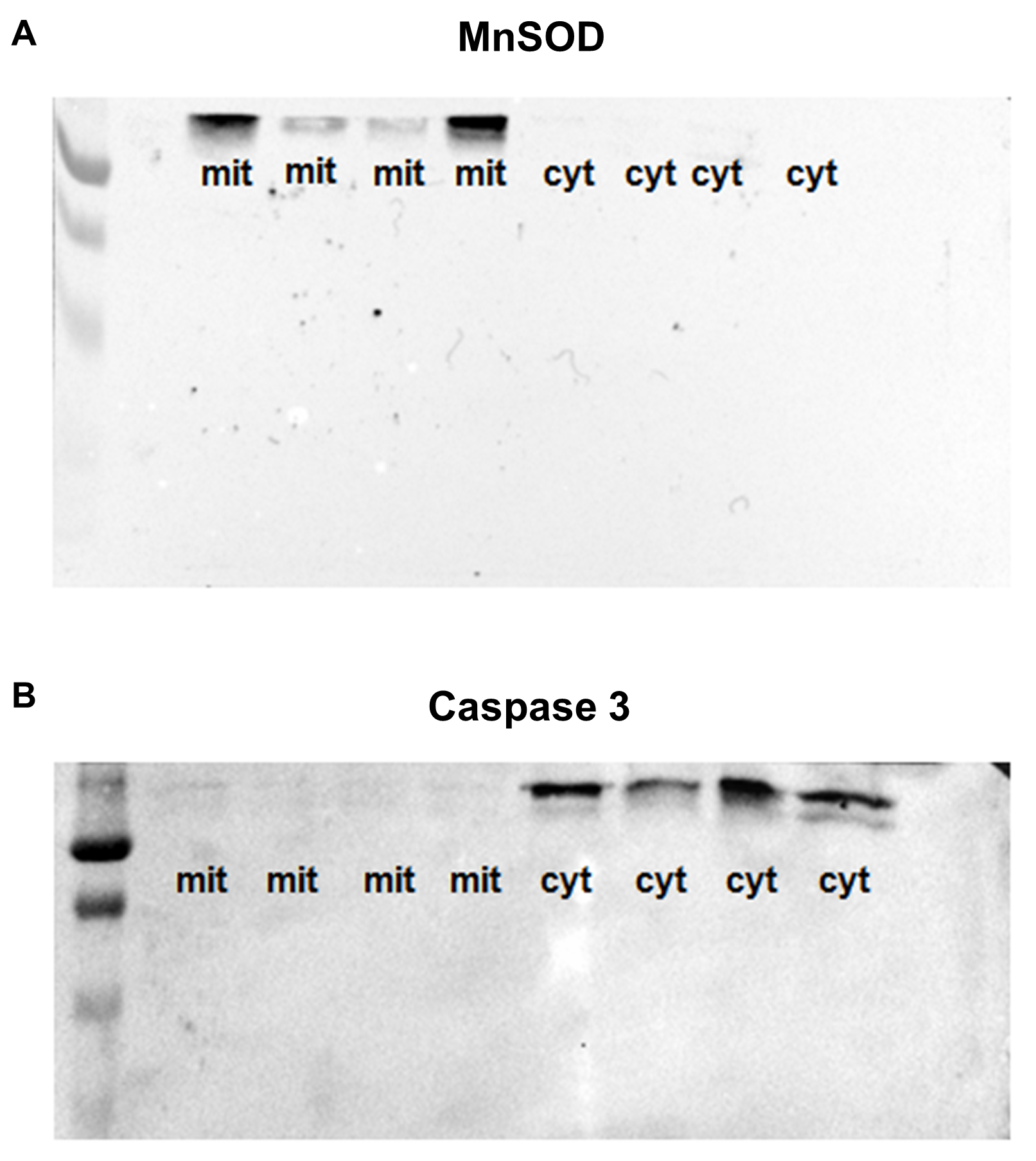

Supplement: Supplementary file 8 — Supporting Information [file CTM2-14-e1654-s002.tif]

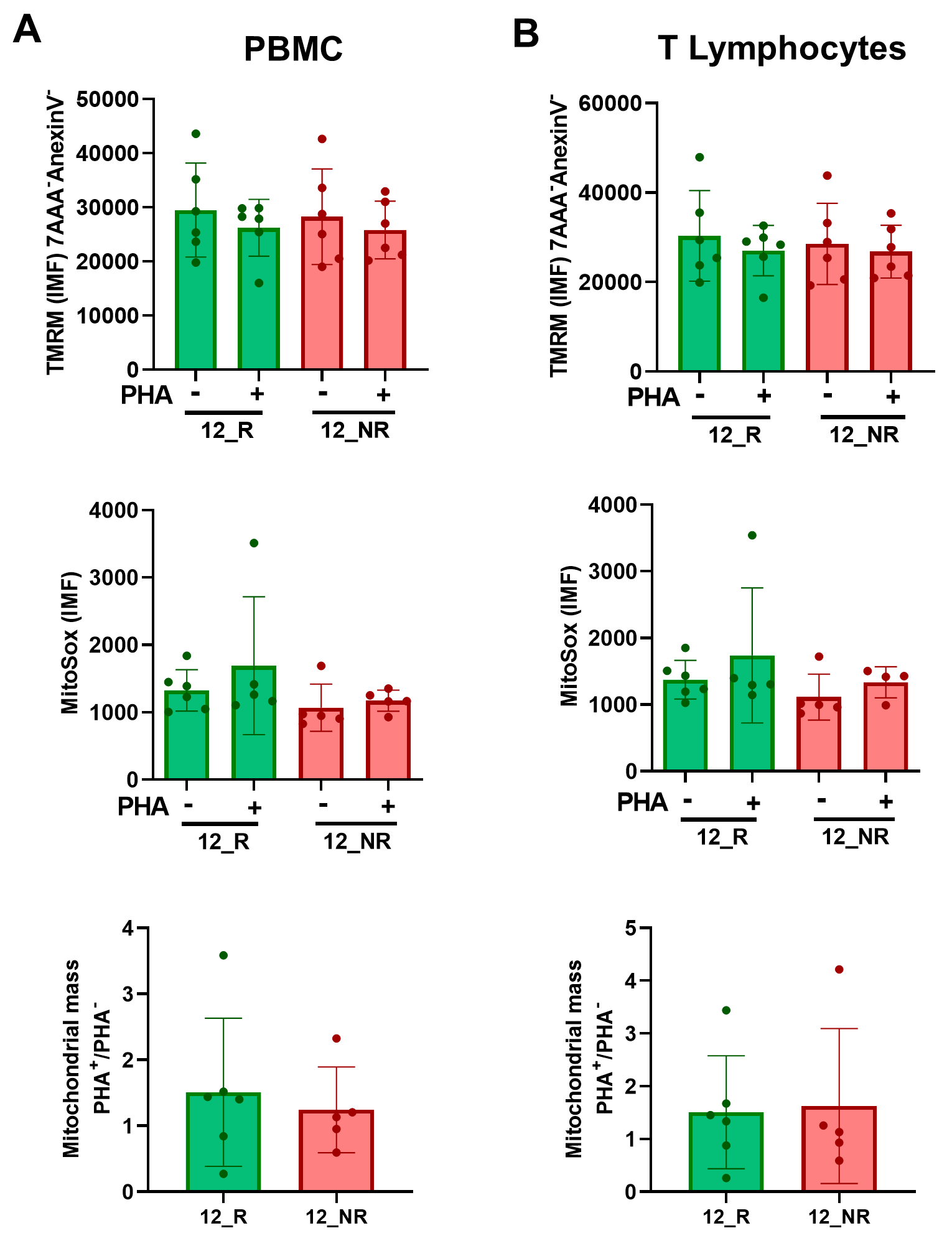

Supplement: Supplementary file 9 — Supporting Information [file CTM2-14-e1654-s010.tif]

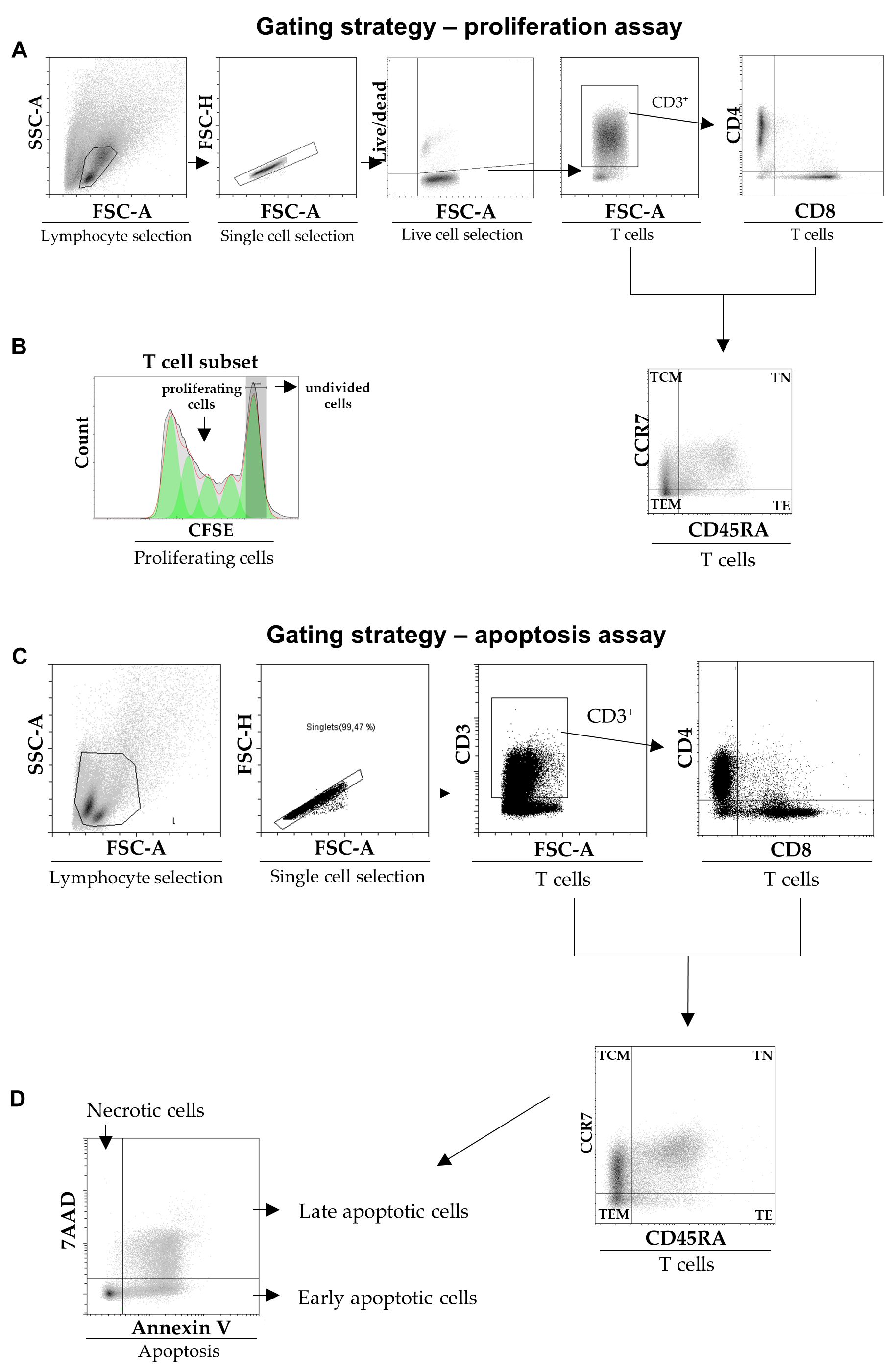

Supplement: Supplementary file 10 — Supporting Information [file CTM2-14-e1654-s011.tif]

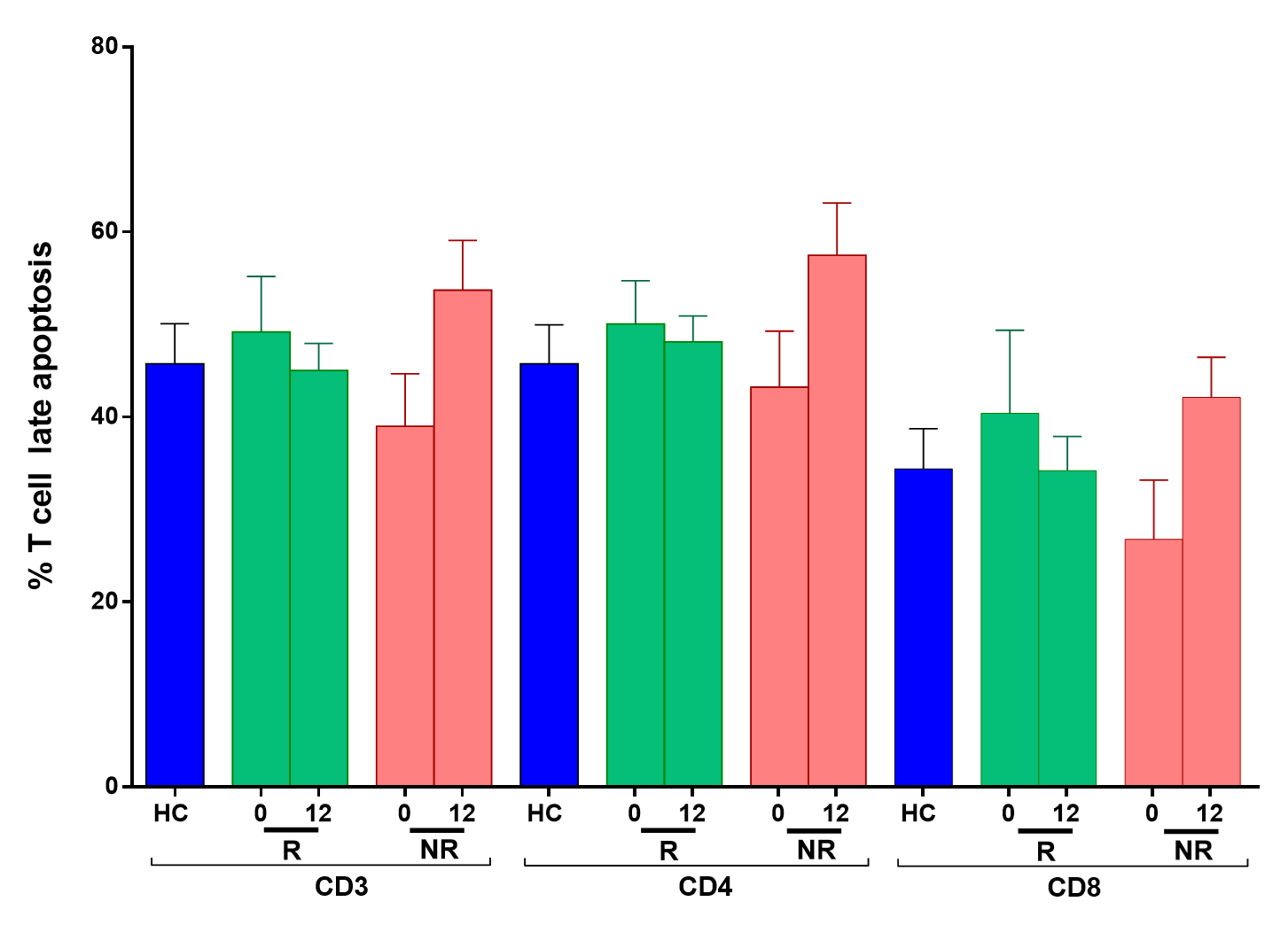

Supplement: Supplementary file 11 — Supporting Information [file CTM2-14-e1654-s006.tif]
